# Non-Gaussian normative modelling with hierarchical Bayesian regression

**DOI:** 10.1162/imag_a_00132

**Published:** 2024-04-25

**Authors:** Augustijn A. A. de Boer, Johanna M. M. Bayer, Seyed Mostafa Kia, Saige Rutherford, Mariam Zabihi, Charlotte Fraza, Pieter Barkema, Lars T. Westlye, Ole A. Andreassen, Max Hinne, Christian F. Beckmann, Andre Marquand

**Affiliations:** Donders Institute for Brain, Cognition and Behavior, Radboud University Nijmegen, Nijmegen, The Netherlands; Department for Cognitive Neuroscience, Radboud University Medical Center Nijmegen, Nijmegen, The Netherlands; Department of Psychiatry, University Medical Center Utrecht, Utrecht, The Netherlands; Department of Psychiatry, University of Michigan, Ann Arbor, MI, United States; MRC Unit for Lifelong Health & Ageing, University College London, London, United Kingdom; Department of Psychology, University of Oslo, Oslo, Norway; Norwegian Centre for Mental Disorders Research, Division of Mental Health and Addiction, Oslo University Hospital and University of Oslo, Oslo, Norway; KG Jebsen Centre for Neurodevelopmental Disorders, University of Oslo, Oslo, Norway; Centre for Functional MRI of the Brain, University of Oxford, Oxford, United Kingdom; Department of Neuroimaging, Institute of Psychiatry, Psychology, & Neuroscience, King’s College London, London, United Kingdom

**Keywords:** normative modelling, hierarchical Bayesian regression, neuroimaging, precision psychiatry

## Abstract

Normative modelling is an emerging technique for parsing heterogeneity in clinical cohorts. This can be implemented in practice using hierarchical Bayesian regression, which provides an elegant probabilistic solution to handle site variation in a federated learning framework. However, applications of this method to date have employed a Gaussian assumption, which may be restrictive in some applications. We have extended the hierarchical Bayesian regression framework to flexibly model non-Gaussian data with heteroskdastic skewness and kurtosis. To this end, we employ a flexible distribution from the sinh-arcsinh (SHASH) family, and introduce a novel reparameterisation and a Markov chain Monte Carlo sampling approach to perform inference in this model. Using a large neuroimaging dataset collected at 82 different sites, we show that the results achieved with this extension are equivalent or better than a warped Bayesian linear regression baseline model on most datasets, while providing better control over the parameters governing the shape of distributions that the approach is able to model. We also demonstrate that the attained flexibility is useful for accurately modelling highly nonlinear relationships between aging and imaging derived phenotypes, which shows that the extension is important for pushing the field of normative modelling forward. All methods described here are available in the open-source pcntoolkit.

## 1 Introduction

Brain disorders affect millions of people worldwide, and have a large impact on the quality of life of the patient and the people surrounding them. Correct treatment in the form of medication or therapy can help reduce symptoms. However, studies to understand mechanisms or develop treatments for these disorders are usually performed in a case-control framework, which provides group-level inferences in which homogeneity within clinical cohorts and clinical groups is presumed. These studies aim to detect differences between the clinical and the control (i.e., healthy) groups and are therefore limited to providing inferences about group averages (e.g., the “average patient”). However, it has been argued that the individual deviations from the groups’ means contain the most important information for stratification (Marquand, Rezek, et al., 2016;Marquand, Wolfers, et al., 2016). An improved understanding of the heterogeneity within clinical cohorts may ultimately lead to better diagnosis and treatment.

Normative modelling provides a framework for understanding heterogeneity within clinical cohorts (Marquand, Rezek, et al., 2016). A normative model aims to provide estimates of centiles of variation of a specific set of phenotypes over the full range of the clinical covariates, that is, across the lifespan, much like growth charts in pediatric medicine. Centiles of variation of imaging derived phenotypes (IDPs) are estimated, and subjects are assigned a point somewhere along the estimated centiles. The assumption is that healthy subjects typically land in areas of higher density than non-healthy subjects. The mean or “average” subject is thus also a meaningful concept in normative models, but contrary to case-control studies, the deviations from that mean are interpreted as anomaly in the brain structure or function, and are seen as valuable resources for further downstream processing like analysis of underlying mechanism of brain disorders.

In probabilistic modelling, a distinction is made between epistemic and aleatoric uncertainty (Kendall & Gal, 2017). The former is due to uncertainty about a parameter or phenomenon, and generally becomes smaller as more data are collected; the latter is uncertainty due to the variation that is naturally present in the measured phenomenon. In normative modelling, we aim to capture and separate these forms of uncertainty to the highest possible degree. Separating all sources of aleatoric and epistemic uncertainty allows adequately controlling for nuisance variation, and guiding further decisions by relevant (biological) variation only.

As in many other fields, data availability in neuroscience has increased dramatically in recent years, and that trend will likely continue. Medical data, however, is sometimes subject to restrictions involving sharing, due to certain permissions not being granted. There is no guarantee that all data on which a model is to be trained can be centrally located. The question of how to do normative modelling on large, distributed datasets is thus a natural one, and it comes with a number of challenges. Firstly, the method must be able to handle large amounts of data. We have seen that Gaussian processes would be viable candidates for normative modelling, if it were not for their time complexity that scales cubically with the number of input data points (Marquand, Rezek, et al., 2016). Second, distributed datasets inevitably come with sources of nuisance variance; since every scan site has its own protocols, every scanner has its own unique noise characteristics, and there may be unknown sources of noise present in specific sites as well. In addition, it is well known that there are sex-related differences in neurobiological phenotypes. Hirearchical Bayesian Regression (HBR) can deal with these so-called batch effects in data by assuming a shared prior over batch-specific model parameters, as we describe inAppendix B. Third, neuroimaging data are complex and provide rich information about the individual from which they were derived. These data therefore need to be treated with care. Permission to share personal data is not always available, and transportation of the data is therefore not always possible. This requires federated learning; a distributed learning approach that does not require transportation of the data, only of the inferred parameters of model (Kia et al., 2021;Li et al., 2020). In this study, we employ and extend the hierarchical Bayesian regression (HBR) approach for normative modelling (Gelman & Rubin, 1992;Kia et al., 2020,^2021^) on non-Gaussian IDPs. The method involves defining a generative model, and inferring the posterior distribution over the model parameters using Markov chain Monte Carlo (MCMC) sampling. By absorbing and summarizing information contained in training data in learned posteriors, HBR supports federated learning; all information required to extend or adapt the model is contained in the model, eliminating the need of data transportation when using or even extending the model.

The current implementation of HBR assumes that the residuals follow a Gaussian distribution. However, the variability in many imaging phenotypes cannot be properly described by a Gaussian distribution, as they are skewed, kurtotic, or both. The issue of non-Gaussianity in neurological phenotypes has partially been addressed inFraza et al. (2021). This method accounts for non-Gaussianity by applying a monotonic warping function to the data (Snelson et al., 2003), which yields a closed form for the marginal likelihood that can be maximised using standard optimisation techniques (Dinga et al., 2021). Here, we utilise the same warping function—the sinh-arcsinh transformation,Appendix A—but in a fully Bayesian approach, that is, in the HBR framework, yielding full posterior distributions for all parameters. Applying the sinh-arcsinh transformation to a standard Gaussian distribution yields the SHASH distribution (Jones & Pewsey, 2009), which is detailed inSection 2.1, a flexible distribution with separate parameters roughly corresponding to skew and kurtosis. Here, we apply the HBR framework to the a canonical variant of SHASH distribution and a novel reparameterisation that substantially reduces dependency between the parameters, which is crucial for more efficient sampling. We assess its performance on some known problematic imaging phenotypes, and show that the HBR framework with a SHASH likelihood is able to model phenotypes in a way that was previously impossible.

The contributions of this work are: (i) a reparameterisation of SHASH distribution and proposal of an MCMC sampling approach to infern this model; (ii) a thorough analysis of the HBR method with a SHASH likelihood in comparison with a warped-Bayesian linear regression as a baseline method (Fraza et al., 2021); (iii) an extensive evaluation of these methods on a large multi-site neuroimaging dataset containing estimates of cortical thickness and subcortical volume; and (iv) an extension to the existing HBR implementation in the pcntoolkit.

The paper is structured as follows: we first provide a theoretical background of HBR, MCMC sampling, and the SHASH distribution inSection 2, where the problems of using the SHASH distribution in a sampling context will also become clear. We introduce a reparameterisation that addresses these problems inSection 2.1.1. A framework for concisely describing common variations of HBR models is introduced inAppendix B. Then, we evaluate our approach using several experiments with a large neuroimaging dataset inSection 3, where we show the proposed method performs on par or better than existing methods. Then, we apply the new HBR models to an even more non-linear dataset (Zabihi et al., 2022), where we show a clear advantage over existing methods. A discussion of the advantages and limitations of our approach follows inSection 4, and we summarise all our findings inSection 5.

## 2 Methods

At its heart, normative modelling involves fitting a probabilistic regression model to predict centiles of variation in a biological or psychometric response variable as a function of a set of clinically relevant covariates. The covariates are often taken to be demographic variables such as age and sex, but this is not necessarily the case and many other mappings are possible (e.g., using cognitive scores to predict brain activity). Early reports used classical Gaussian process regression (Marquand, Rezek, et al., 2016;Ziegler et al., 2014) which is appealing due to its Bayesian non-parametric nature, exact inference and flexibility in modelling non-linearity with a relatively small number of parameters. However, the exact Gaussian process approach used in these works has two main limitations, namely its extremely poor computational scaling to large numbers of data points (owing largely to the cubic computational scaling required to solve the GPR predictive equations) and for the exact formulation (i.e., having an analytical solution), it is restricted to modelling Gaussian centiles. While generalisations are possible within the Gaussian process literature to address these problems, these typically have other shortcomings. For example, modelling non-Gaussian noise distributions usually requires approximations which further increases computational complexity. The result is that other approaches with better computational scaling have been proposed in the neuroimaging literature. These are reviewed inMarquand et al. (2019)and include quantile regression and hierarchical linear regression techniques. More recently, the field has increasingly recognised the importance of modelling non-Gaussianity in the response variables, while using a distributional form to increase the precision with which outer centiles can be estimated (Borghi et al., 2006). There are two main approaches that have been proposed to solve this problem in the neuroimaging literature, namely likelihood warping (Fraza et al., 2021) and generalised additive models for location, scale, and shape (GAMLSS) (Dinga et al., 2021). While these approaches are both capable of flexibly modelling non-Gaussianity and site effects, both have potential shortcomings: GAMLSS is not probabilistic due to the heavy regularisation penalties that are applied to constrain the flexible smoothers underlying the approach. Likelihood warping is probabilistic in nature, but still does not model the uncertainty associated with shape parameters and does not offer completely flexible control over the nature of the modelled distribution. Neither approach supports fully decentralised federated learning at training time although ad-hoc solutions have been proposed to allow model parameters to be transferred to a new site at test time. The approach proposed in this paper attempts to address these shortcomings.

The HBR framework is a Bayesian modelling framework that assumes a generative model over the response variablesYin the form of a likelihoodℒand a prior distributionpfor each parameter$\theta_{i}$of the likelihood. A general model can be expressed as such:

$$\begin{array}{ccccccc} \theta_{i}∼p_{\theta_{i}} & & i\in \left\{1,...,P\right\} & & & & \\ Y∼ℒ(Y|\left\{\theta_{i}\right\}), & & & & & & \end{array}$$ (1)

wherePis the number of parameters of the likelihood. One of the main objectives in Bayesian modeling is to retrieve the posterior, which is the distribution over the model parameters given the data;p(θ|Y). Bayes’ rule gives us an expression of the posterior:p(θ|Y)=p(Y|θ)p(θ)/p(Y), but this expression can only be evaluated analytically in special cases where the likelihood is conjugate to the prior, and this is not the case in HBR. As of such, the posterior is approximated by MCMC sampling, which we discuss inAppendix D. Assuming the response variable follows a Gaussian distribution, we substitute the GaussianNforℒ, and our parametersθbecome the mean and varianceμand$\sigma^{2}$. The general model described inEquation 1is fully capable to support non-Gaussian likelihoods, like the family of SHASH distributions, which is detailed inSection 2.1. The way we model$p_{\theta_{i}}$depends on our further assumptions about the data. Typicallyμ, and optionally alsoσare taken to be linear functions of a set of covariates, which in this example are clinical or demographic variables. This is further detailed inAppendix B, where we also introduce a framework for model specification.

### 2.1 The SHASH distribution

**Fig. 1.**
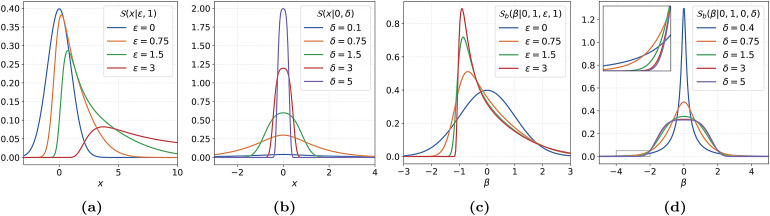
The effects of∈andδon the shape of the SHASH and SHASH*_b_*densities. In (a) and (c), the value of∈was varied whileδwas kept fixed at 1. In (b) and (d), the value ofδwas varied while∈was kept fixed at 0. The densities in (c) and (d) have zero mean and unit variance. The inset in (d) corresponds to the area in the gray box in the bottom left corner of (d).

To accurately fit normative models on non-Gaussian-distributed response variables, one could substitute a family of flexible distributions forℒinEquation 1. This family must then contain positively as well as negatively skewed members, leptokurtic as well as platykurtic members, and preferably with smooth transitions between them. All those requirements are fulfilled by the family of SHASH distributions (S).Figure 1aand1bshow the flexibility of theSin modelling various distributional forms.

To generate samples from a SHASH distribution, one applies an inverse sinh-arcsinh transformation to samples from a standard Gaussian (Jones & Pewsey, 2009). The sinh-arcsinh transformationξand its inverse$\xi^{-1}$are defined as:

$$\begin{array}{cccc} \xi_{\in ,\delta }(x)=\text{sinh(}\delta {\text{sinh}}^{-1}(x)-\in \text{)} & & & \\ \xi_{\in ,\delta }^{-1}(x)=\text{sinh(}({\text{sinh}}^{-1}(x)+\in )/\delta \text{)} & & & \end{array}$$

where∈∈ℝand$\delta \in {\mathbb{R}}^{+}$govern the shape of the resulting distribution. Now assuming a random variableZfollows a standard Gaussian, we can construct samplesXthat follow a SHASH distribution by applying$\xi_{\in ,\delta }^{-1}$toZ^1^:

$$\begin{array}{cccc} Z∼N\left(0,1\right) & & & \\ X=\xi_{\in ,\delta }^{-1}\left(Z\right) & & & \\ X∼S\left(\in ,\delta \right) & & & \end{array}$$ (2)

For the remainder ofSection 2,Zandzwill indicate Gaussian distributed samples, andXandxwill indicate SHASH distributed samples.

To derive the density of this SHASH distribution, we need to apply a change of variables, which states that the density of transformed samples will be the density of the original samples multiplied with the Jacobian determinant of the inverse transformation (Bishop, 2006). The constraint being that this Jacobian determinant exists and that it is not 0. This is true for any transformation that is a diffeomorphism^2^. Because the transformationξis a diffeomorphism, we can get the density ofSby a change of variables. We multiply the density of the samples in the Gaussian domain,φ,—which we find by applyingξ—with the derivative of$\xi_{\in ,\delta }$.

$$\begin{array}{l} S\left(x|\in ,\delta \right)=\frac{\partial \xi_{\in ,\delta }}{\partial x}\varphi (\xi_{\in ,\delta }(x)|0,1)\\ \;\;\;\;\;\;\;\;\;\;\;\;\;\;\;\;\;\;\;\;\;\;\;\;\;=\frac{\delta C_{\in ,\delta }(x)}{\sqrt{1+x^{2}}}\frac{1}{\sqrt{2\pi }}\text{exp}\left(\frac{-\xi_{\in ,\delta }{\left(x\right)}^{2}}{2}\right), \end{array}$$ (3)

where$C_{\in ,\delta }(x)=\text{cosh(}\delta {\text{sinh}}^{-1}(x)-\in \text{)}$.Figures 1and2illustrate the effect of the parameters∈andδon the shape of the SHASH density. We clearly see that∈andδmodulate the skew and kurtosis, respectively.

**Fig. 2.**
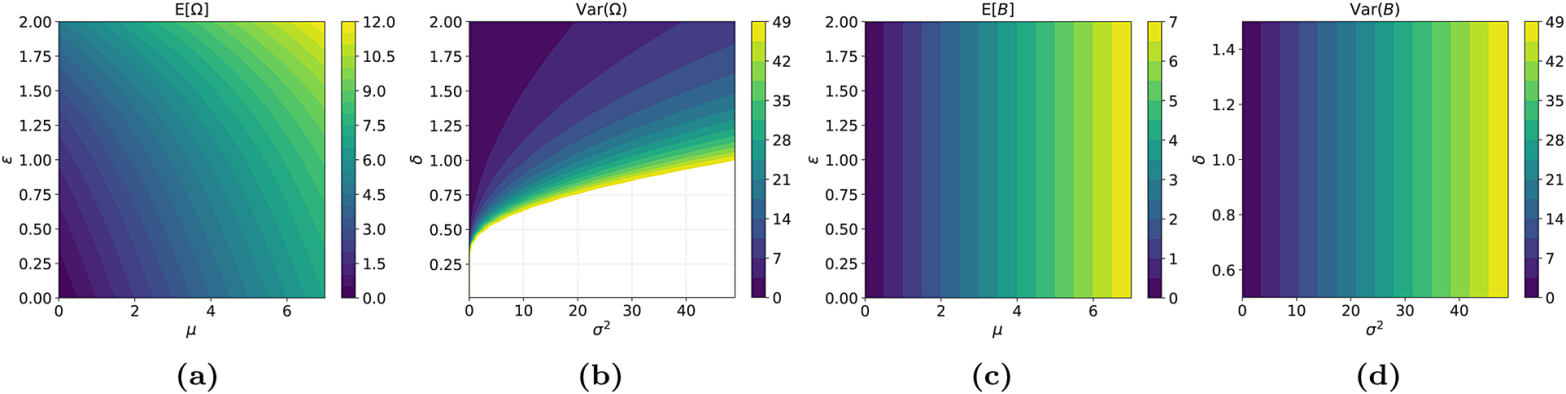
Correlations in the parameter space. In (a), at∈=0, the mean is exactly the value ofμ, but when∈≠0, that is not the case. Similarly, in (b), the variance is exactly$\sigma^{2}$only on the lineδ=1. (c) and (d) Show the parameter space under the proposed reparameterisation. The correlation between these pairs of parameters is now removed.

A constraint on the parameterδcan be derived from this result. A well-known property of probability distributions is that they are positive everywhere. The only term inEquation 3that can possibly be negative is the left fraction, because the middle fraction is a positive constant and the right exponential is strictly positive for all real inputs^3^. The left fraction is negative if exactly one ofδor$C_{\in ,\delta }$is negative, and we havecosh(α)>0for allα∈ℝ. Thus, the only way in which we can get a negative density is to haveδ<0, from which we derive the constraint thatδ>0.

#### 2.1.1 Reparameterisation

The SHASH distribution we have seen so far has two parameters,∈andδ, but additional parameters of location and scale are necessary to achieve the desired flexibility.Jones and Pewsey (2009)suggest adding scale and location parameters by muliplication withσand addition ofμ. The resulting distribution is known as the SHASH*_o_*or$S_{o}$distribution.

Assuming a random variableZfollows a standard Gaussian, we can construct samplesΩthat follow a$S_{o}$distribution by applying$\xi_{\in ,\delta }^{-1}$toZ, multiplying withσ, and addingμ:

$$\begin{array}{cccc} \text{Ω}=\xi_{\in ,\delta }^{-1}\left(Z\right)\sigma +\mu & & & \\ \text{Ω}∼S_{o}\left(\mu ,\sigma ,\in ,\delta \right) & & & \end{array}$$

Equivalently, because of2, we can write

Ω=Xσ+μ (4)

And because we already know the density ofXto beS, we can use the chain rule in conjunction with the change of variables to arrive at the density of$S_{o}$:

$$S_{o}(\omega |\mu ,\sigma ,\in ,\delta )=S((\omega -\mu )\sigma^{-1}|\in ,\delta )\sigma^{-1}.$$ (5)

where∈andδare parameters that govern the higher order moments of the distribution (explained below). While we believe this family is flexible enough for the purposes of modeling a wide variety of neuroimaging phenotypes, inferring the parameters of this distribution is highly challenging because of their strong correlations^4^.Figures 1aand2aillustrate the correlation betweenμand∈. Clearly,∈controls both the skew and the mean, butμalso controls the mean. Similarly, inFigures 1band2b, we see thatδcontrols the kurtosis as well as the variance, whileσalso controls the variance. Writing down the first two moments of the$S_{o}$distribution will make this correlation explicit. Because the samplesΩfrom SHASH*_o_*are simply scaled and shifted samplesXfrom SHASH (seeEquation 5), we can express the moments ofΩsimply in terms of the moments ofXas:

E[Ω]=μ+σE[X] (6)

$$\text{Var}\left(\text{Ω}\right)=\sigma^{2}\text{Var}\left[X\right].$$ (7)

To break the correlation betweenμand∈and betweenσandδ, we propose to use the expressions forE [X]and$E\;[X^{2}]$that are provided by Jones et al. to derive a new density. Jones et al. give us an analytical expression for ther’th non-central moment, given here inEquation 8. For derivations of these quantities, please refer toJones and Pewsey (2009).

$$\begin{array}{l} m_{\in ,\delta }^{\left(r\right)}=E[X_{\in ,\delta }^{r}]=\frac{1}{2^{r}}\sum_{i=0}^{r}\left(\begin{array}{c} r\\ i \end{array}\right){\left(-1\right)}^{i}\text{exp}\left(\left(r-2i\right)\frac{\in }{\delta }\right)\\ \;\;\;\;\;\;\;\;\;\;\;\;\;\;\;\;\;\;\;\;\;\;\;\;\;\;\;\;\;\;\;\;\;\;\;\;\;\;\;\;\;\;\;\;\;P\left(\left(r-2i\right)/\delta \right), \end{array}$$ (8)

forr∈ℕ, where

$$P\left(q\right)=\frac{e^{1/4}}{{\left(8\pi \right)}^{1/2}}\left(K_{\frac{q+1}{2}}\left(\frac{1}{4}\right)+K_{\frac{q-1}{2}}\left(\frac{1}{4}\right)\right),$$ (9)

andKis the modified Bessel function of the second kind (Bowman, 1939).

The solution proposed here is to first apply a standardising shift and scale to the SHASH samples, such that the mean and variance of those samples are 0 and 1, respectively. Then, we apply the location and scale parametersμandσlike before to attain samplesB. Because the moments ofSare all obtainable byEquation 8, this is a simple operation. We define the newly designed transformation asλand the corresponding density as SHASH*_b_*or$S_{b}$:

$$\begin{array}{cccc} \lambda_{\mu \sigma \in \delta }\left(z\right)=\left(\xi_{\in \delta }^{-1}\left(z\right)-m_{\in \delta }^{\left(1\right)}\right){\left(\eta_{\in ,\delta }^{2}\right)}^{-\frac{1}{2}}\sigma +\mu . & & & \\ B=\lambda_{\mu \sigma \in \delta }\left(Z\right) & & & \\ B∼S_{b}\left(\mu ,\sigma ,\in ,\delta \right) & & & \end{array}$$ (10)

where$\eta_{\in ,\delta }^{2}$is the central variance given by$\eta_{\in ,\delta }^{2}=m_{\in ,\delta }^{\left(2\right)}-$${\left(m_{\in ,\delta }^{\left(1\right)}\right)}^{2}$. We can again use the chain rule in conjunction with the change of variables to arrive at the density of$S_{b}$:

$$\begin{array}{ccc} S_{b}\left(\beta |\mu ,\sigma ,\in ,\delta \right)=S\left(\left(\beta -\mu \right)\eta_{\in ,\delta }\sigma^{-1}+m_{\in ,\delta }^{\left(1\right)}|\in ,\delta \right)\eta_{\in ,\delta }\sigma^{-1}, & & \end{array}$$ (11)

Writing down the first and second moments ofB, the expectations that appeared inEquations 6and7now cancel with the moments that we added inEquation 10, and the correlation breaks completely:

E[B]=μ (12)

$$\text{Var}\left(B\right)=\sigma^{2}.$$ (13)

The new parameterisation has nicely interpretable parameters for location and scale, which the$S_{o}$does not. CompareFigure 1aand1bwith 1c and 1d. The densities in the two rightmost figures all have zero mean and unit variance. This may not seem obvious for the density withδ=0.4inFigure 1d, but the tail behaviour responsible for this becomes clearer from the inset. InAppendix G,H, the empirical moments of the$S_{o}$and$S_{b}$distributions are plotted over a range of parameters. It is important to note that the$S_{b}$family is isomorphic to$S_{o}$, which means that they model exactly the same set of distributions, just under a different parameterisation.

#### 2.1.2 Priors on $S_{b}$ parameters

The$S_{b}$reparameterisation greatly reduces the problem of correlation in parameter space, but it comes at a cost. Some of the reparameterisation terms get extremely large quickly, and may therefore lead to numerical instability. This may be addressed by appropriately setting the priors for the$S_{b}$likelihood. ComparingFigure 2band2d, the warp in the low domain ofδis apparent. The stretching effect of the change of variables is enormous in those regions, because it has to compensate for the flattening effect ofδvisible inFigure 1bin order to standardise the SHASH distribution. The curve in the line associated withδ=0.1is barely visible in this range. The variance of$X_{\in ,\delta }$as a function ofδis shown on a double log scale inFigure 3. Notice that small differences in the lower domain ofδamount to substantial differences in the variance. The enormous magnitude of$\frac{d}{d\delta }m_{\in ,\delta }^{\left(2\right)}$in those regions is mainly due to the behaviour of the Bessel function$K_{n}(z)$inEquation 9, and to a lesser degree due to the fraction in the exponent inEquation 8. In practical applications, values ofδ<0.3are thus associated with numerical instability, and sampling in those regions should be avoided at the cost of losing some expressivity. In our models, we enforce this constraint by first enforcing positivity of the samples by applying the softplus function, and then adding a small constant of 0.3. The softplus function enforces positivity and is defined as:

**Fig. 3.**
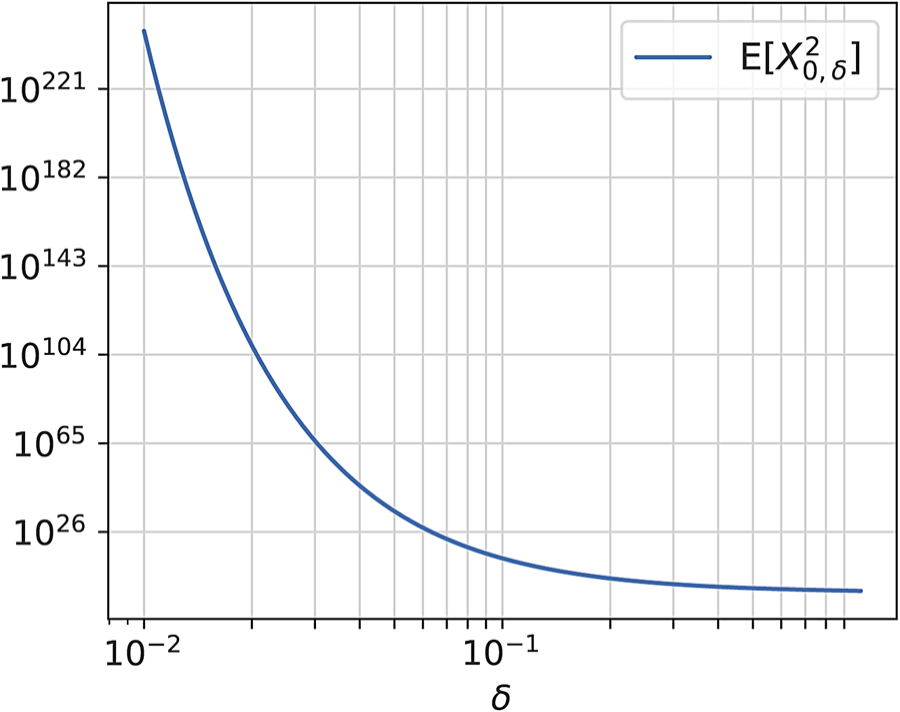
The variance of$X_{\in ,\delta }$forδ<1.

softplus(x)=log(1+exp(x)). (14)

### 2.2 Experiments

#### 2.2.1 Dataset 1: Lifespan normative modelling of image derived phenotypes

To allow a meaningful comparison with the current methods, we adapt and use the data from (Rutherford et al., 2022). This is a large neuroimaging dataset collected from 82 different scanners, containing 58834 control subjects and 2925 patient subjects^5^. Measures of cortical thickness and subcortical volume were extracted with Freesurfer version 6.0 using image derived phenotypes derived either from the Destrieux cortical parcellation or from the subcortical segmentation. Furthermore, the different UK-Biobank datasets (Sudlow et al., 2015) were merged, because based on prior work (Fraza et al., 2021;Kia et al., 2022), we have observed that site effects in the UKB cohort are minimal for these freesurfer measure derived from the different scanners used (due to a careful harmonisation of acquisition parameters and scanning hardware across this cohort). We therefore consider that we can safely consider them to be drawn from a single site. Counts per site and sex can be found inAppendix F. For full details about the data, we refer toRutherford et al. (2022). Most data in this dataset are publicly available, but for several of the clinical samples, we do not have explicit permission for data sharing. Such permissions for sharing were not obtained from the data from the University of Michigan, and as such these data were not included in this study (n = 1394 (1149 controls, 245 patients)).

The family of SHASH distributions has the flexibility to model skewed and kurtotic distributions, but also has the standard Gaussian as a central member. We pick some “hard” (i.e., difficult) phenotypes, the age-conditioned distributions of which are not described well by a Gaussian, and some “easy” phenotypes, the age-conditioned distributions of which are described well by a Gaussian. We aim to model both easy and hard phenotypes with the same model, using the same hyperparameters. The selected hard phenotypes are: right cerebellum white matter, because it is slightly skewed; right lateral ventricle, because it is moderately skewed and heteroskedastic; and white matter hypointensities, because it is severely skewed and heteroskedastic. For easy phenotypes, we selected the estimated total intracranial volume, the thickness of the Jensen sulcus, and the volume of the brain stem. These “easy” phenotypes do not show a lot of skew or kurtosis, but the mean of the thickness of the Jensen sulcus shows an interesting trajectory (seeFig. I.26). The distributions of the different phenotypes are shown inFigure 6and inAppendix Ibelow. In some instances, we used the following abbreviations for the “hard” phenotypes: Right-Cerebellum-White-Matter: RCWM, Right-Lateral-Ventricle: RLV, WM-hypointensities: WMH, and similarly for the “easy” phenotypes: EstimatedTotalIntraCranialVol: ETICV, rh_S_interm_prim-Jensen_thickness: RIPJ, Brain-Stem: BS,

A stratified 10-fold split was made using the Sklearn package (Pedregosa et al., 2011), to which end we filtered out data from sites with less than 10 subjects of one or either sex. This ensures that the test folds contain at least one unique sample for each batch effect. As preprocessing, all data were feature-wise-normalised by subtracting the mean and dividing by the standard deviation. Normalisation is not a necessity, but it allowed the same hyperparameters to be used for different response variables. This would otherwise not be possible, because the scale of the response variables differ by several orders of magnitude. Sampling performance could be hampered if the prior distribution differs exceedingly much from the likelihood.

#### 2.2.2 Batch effects

Age and sex were used as covariates, and site was used as a batch effect. The HBR framework supports multidimensional batch effects, allowing to use both sex and site as a batch effect and allowing site effects to be modelled in a single-step regression together with effects of interest. However, we consider that there is little reason to assume that the differences related to sex vary much between sites, and having this multidimensional batch effect results in a larger number of model parameters. In practice, we saw no difference in performance between models that used sex as a batch effect, and those that used sex as a covariate. For further discussion, seeSection 4.

#### 2.2.3 Dataset 2: Modelling latent representations derived from an autoencoder

As a second, more challenging, test of the flexibility of our model, we fit a normative model on a highly non-linear representation of brain data derived from the latent representation learned by a convolutional autoencoder. Full details are provided inZabihi et al. (2022), but briefly; data derived from the UK Biobank sample (n = 20,781, 47% female) were compressed to a 2-dimensional latent representation using a pipeline consisting of a conditional convolutional autoencoder with a 100-dimensional latent space (Kingma & Welling, 2013), followed by a UMAP dimensionality reduction (McInnes et al., 2018). The two-step pipeline was constructed because the intermediate representation produced by the autoencoder would otherwise not be reflective of age and sex, it would only reconstruct the data. In this case, it was desirable to have a latent representation conditioned on these variables. The resulting representation followed a distribution with a nonlinear trajectory with respect to age n the mean, variance, and skew. A stratified train/test (75%/25%) split was made on the raw data level, specifically not at the 100-dimensional latent representation (seeFig. 9). This means that the same train data were used for training the convolutional autoencoder as well as the UMAP embedding, and all of the test data were held out from training. Following the rationale described above, no batch effects were modelled. Again, we used age and sex as covariates.

#### 2.2.4 MCMC samplers

We employed a No U-Turn sampler for inference, implemented within the PyMC version 5.4.1 software using two chains per sample. Chains were run for 1500 iterations using 500 samples as burn-in. Convergence checks were performed using the$\hat{R}$statistic. Further details about the theory behind MCMC inference are provided inAppendix D. All scripts necessary for running the analyses conducted in this paper are freely available via GitHub.

#### 2.2.5 Ethics statement

All participants provided written informed consent for their data to be used, and all studies were approved by the institutional review boards at the respective institutions.

## 3 Results

InSection 3.1, the convergence of the MCMC samplers utilised by the HBR models is assessed.

We evaluate four variants of the HBR method: The first uses a simple Gaussian likelihood, whereμandσare modelled exactly likeμandσinAppendix B.3. This model is indicated with anN. ModelNis the model that was previously the limit of what could be done within HBR in the pcntoolkit. We also evaluate a model with an$S_{o}$likelihood and two variants of the$S_{b}$likelihood, which differ only in the way∈andδare modelled. In the first model$S_{b1}$, both∈andδassume constant values throughout the full range of covariates. In model$S_{b2}$, both∈andδare linear regressions on the design matrixΦ, which in our case contains 5-knot b-spline basis expansion (Boor, 1978) of the age covariate, concatenated with the age and sex covariates. These models are formalised inTable 1, using the notation detailed inAppendix B. Details about the priors can be found inTable 2. Full specifications of the generative models can be found inAppendix C.

**Table 1.** The generative descriptions of the models that are compared to W-BLR, using the notation detailed inAppendix B.

N	$S_{o1}$	$S_{b1}$	$S_{b2}$
$\mu_{n}∼{ℳ}_{3.1.2b}$	$\mu_{n}∼{ℳ}_{3.1.2b}$	$\mu_{n}∼{ℳ}_{3.1.2b}$	$\mu_{n}∼{ℳ}_{3.1.2b}$
$\sigma_{n}^{\pm }∼{ℳ}_{3.1.1}$	$\sigma_{n}^{\pm }∼{ℳ}_{3.1.1}$	$\sigma_{n}^{\pm }∼{ℳ}_{3.1.1}$	$\sigma_{n}^{\pm }∼{ℳ}_{3.1.1}$
$\sigma_{n}=\text{softplus(}\sigma_{n}^{\pm }\text{)}$	$\sigma_{n}=\text{softplus(}\sigma_{n}^{\pm }\text{)}$	$\sigma_{n}=\text{softplus(}\sigma_{n}^{\pm }\text{)}$	$\sigma_{n}=\text{softplus(}\sigma_{n}^{\pm }\text{)}$
$y_{n}∼N(\mu_{n},\sigma_{n})$	$\in ∼{ℳ}_{1}$ $\delta^{\pm }∼{ℳ}_{1}$	$\in ∼{ℳ}_{1}$ $\delta^{\pm }∼{ℳ}_{1}$	$\in ∼{ℳ}_{3.1.1}$ $\delta^{\pm }∼{ℳ}_{3.1.1}$
	$\delta =\text{softplus(}10\cdot \delta^{\pm }\text{)}/10$	$\delta =\text{softplus}(10\cdot \delta^{\pm })/10$	$\delta =\text{softplus}(10\cdot \delta^{\pm })/10$
	$y_{n}∼S_{o}(\mu_{n},\sigma_{n},\in ,\delta +0.3)$	$y_{n}∼S_{b}(\mu_{n},\sigma_{n},\in ,\delta +0.3)$	$y_{n}∼S_{b}(\mu_{n},\sigma_{n},\in ,\delta +0.3)$

For alln,n∈{1,...,N}. The softplus function was scaled down by a factor of 10 to force more linear behaviour between 0 and 1. More details about the priors can be found inTable 2. The notation${ℳ}_{a.b.c}$is used to denote different generative models, the details of which can be found inAppendix C.

**Table 2.** Prior settings for all HBR models used in this report.

Parameter	Prior	N	$S_{o1}$	$S_{b1}$	$S_{b2}$
${\text{w}}_{\mu }$	$N({\text{w}}_{\mu }|0_{D},{\text{I}}_{D})$	X	X	X	X
$\mu_{\tau_{\mu }}$	$N(\mu_{\tau_{\mu }}|0,1)$	X	X	X	X
$\sigma_{\tau_{\mu }}$	$N^{+}(\sigma_{\tau_{\mu }}|1)$	X	X	X	X
$\nu_{\tau_{\mu }}$	$N(\nu_{\tau_{\mu }}|0,1)$	X	X	X	X
${\text{w}}_{\sigma^{\pm }}$	$N(\mu_{{\text{w}}_{\sigma^{\pm }}}|0_{D},{\text{I}}_{D})$	X	X	X	X
$\tau_{\sigma^{\pm }}$	$N(\tau_{\sigma^{\pm }}|1,1)$	X	X	X	X
∈	N(∈|0,1)		X	X	
${\text{w}}_{\in }$	$N({\text{w}}_{\in }|0_{D},0.2\cdot {\text{I}}_{D})$				X
$\tau_{\in }$	$N(\tau_{\in }|0,0.2)$				X
$\delta^{\pm }$	$N(\delta^{\pm }|1,1)$		X	X	
${\text{w}}_{\delta }$	$N({\text{w}}_{\delta }|0_{D},0.2\cdot {\text{I}}_{D})$				X
$\tau_{\delta }$	$N(\tau_{\delta }|1,0.3)$				X

Note that parameters with a±superscript are mapped by a softplus function post-sampling to force positivity, as displayed inTable 1.$N^{+}$indicates the positive Normal distribution.$0_{D}$and$I_{D}$denote aDdimensional zero-vector and identity matrix, respectively. Central values were chosen for most priors, withδbeing a notable exception. As discussed before, having a lowδleads to numerical issues, and by setting the priors up like this, we aim to push the sampler away from the low domain. Remember that we also add a constant of 0.3 to the result of applying the softplus function to$\delta^{\pm }$. The prior on$\tau_{\sigma }$is centred at 1, because due to standardisation we expectσto lay close to 1 on average, and the softplus approximates an identity function for positive values, so the mode of the transformed value should still lay close to 1. We set the prior on the weights ofμa little wider than the others, because we found that improved the regression. Making the priors any wider than this did not lead to any significantly different results.

InSection 3.2, we compare different types of HBR normative models, with one another and with warped Bayesian Linear Regression (Fraza et al., 2021), which is used as a baseline, abbreviated with W-BLR. The models used inSection 3.3are the same as inSection 3.2, just applied to different data.

Note that the results inSection 3.2are based on MCMC estimates of z-scores (seeAppendix E). The results inAppendix Care based on the MAP estimates, which were chosen because they are less computationally demanding, and because in practice, the MCMC results were only marginally better.

A main objective in normative modelling is to map observations to z-scores in such a way that healthy variation within those z-sores follows a Gaussian distribution, and that patients fall in the outer centiles of variation. Gaussianity in the outer centiles is compared by the third and fourth moment (skew and kurtosis) of the predicted z-scores. The qq-plots, and a visual inspection of the regression curves further help assess the centiles.

### 3.1 Convergence analysis

Here a simple analysis of the samplers is performed, by analysing the trajectory of the Gelman-Rubin, or$\hat{R}$statistic (Brooks & Gelman, 1998), which can be thought of as the ratio between the interchain variance and the intrachain variance. The$\hat{R}$statistic is 1 (one) when those are equal, and this is a good indicator that the sampler has converged. In practice, a rule of thumb is that an$\hat{R}$lower than 1.1 is no reason to worry, and an$\hat{R}$less than 1.05 indicates good convergence. To compute the$\hat{R}$statistic, at least two chains need to be sampled. We sampled two chains of 1500 samples using the pcntoolkit, the first 500 of which were used for tuning and were discarded. The last 1000 samples of all chains were stored and used for further analysis.

InFigure 4, the$\hat{R}$statistics of∈(unbroken line) andδ(broken line) are displayed as a function of chain length. The results are averaged over the 10 folds. Overall, the convergence of the$S_{o1}$model, the$S_{b1}$model, and the$S_{b2}$models are similar on these phenotypes. Except for the WM-hypointensities phenotype—which is very hard to model—all samplers converge with$\hat{R}$very close to 1 within at most 300 iterations.

**Fig. 4.**
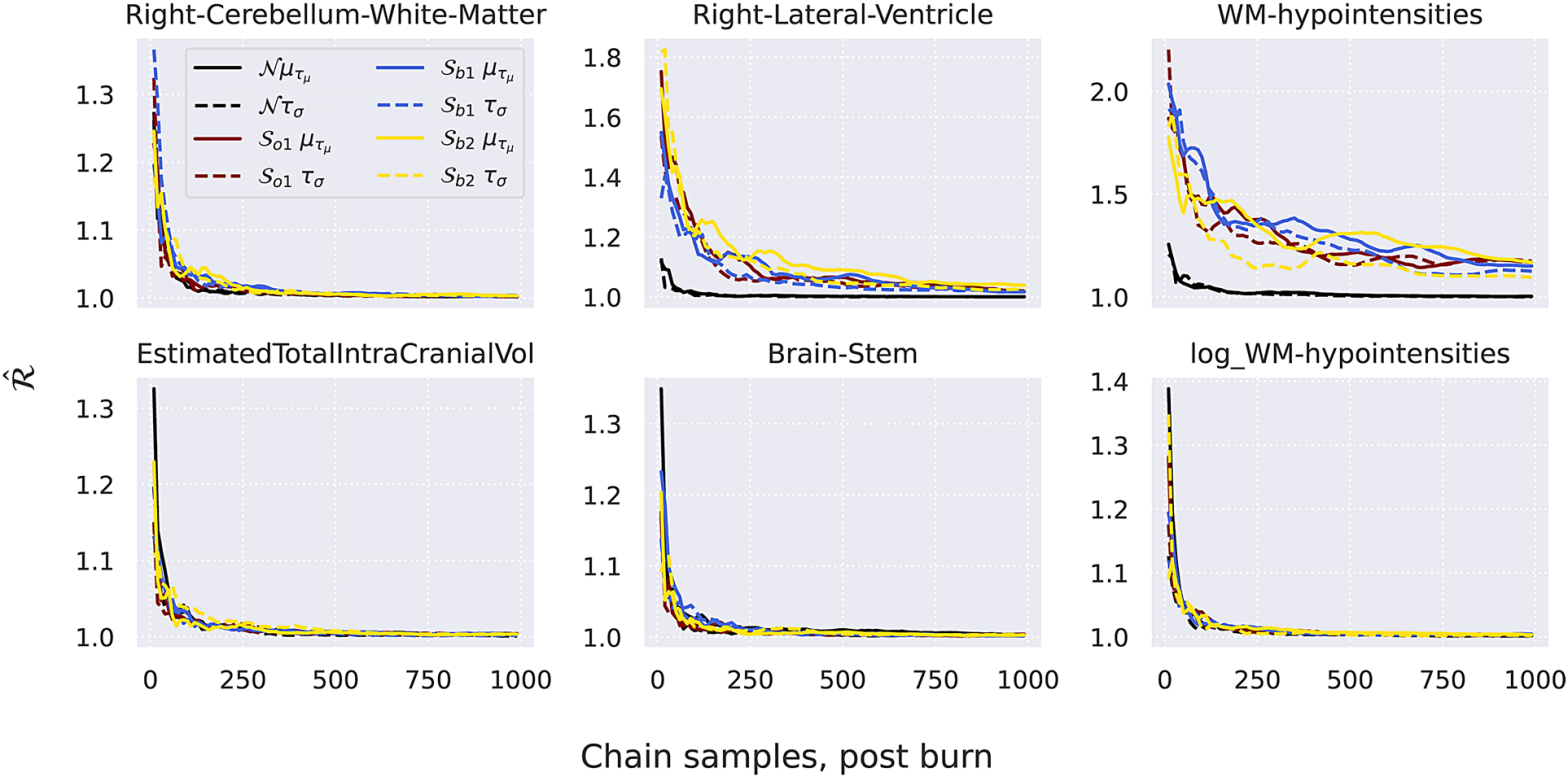
$\hat{\text{R}}$of∈andδfor$S_{o}$,$S_{b1}$, and$S_{b2}$, average of 10 folds. For the selection of “easy” phenotypes in this panel, all models behave comparably. For the selection of “hard” phenotypes, the$S_{o}$,$S_{b1}$,$S_{b2}$models behave comparably. “Hard” phenotypes: Right-Cerebellum-White-Matter, Right-Lateral-Ventricle, (log)White-Matter-Hypointensities. “Easy” phenotyopes: Estimated-Total-Intracranial-Volume, Brain-Stem. See alsoSection 2.2.

Since it is a strictly positive measure, we also evaluate the convergence of the samplers on a log-transformed version of the WM-hypointensities, which shows much better convergence.

Note also that the$S_{o}$sampler did not converge for the most difficult phenotype reported inSection 3.3, so we use the$S_{b}$parameterisation in that case.

### 3.2 Goodness-of-fit analysis

First, we discuss how the HBR with the$S_{b}$likelihoods compare to the W-BLR method, and the HBR with the Gaussian likelihood. We assess (i) how well the predicted z-scores follow a Gaussian distribution, by looking at the higher-order moments of the z-scores and at qq-plots; (ii) how good the fit of the percentile lines is visually, by superimposing them on the data; and (iii) how well the models have captured the batch effects, by looking at classification performance. Specifically: for any combination of two sites, we computed the area-under-the-curve (AUC) metric (Hastie et al., 2009) by using the z-scores as classification thresholds, and the batch-indices as labels.

Table 3forms the first basis of our discussion. For convenience, it is useful to juxtaposeFigure 6orAppendix I, which visualise the phenotypes discussed here. The skew and kurtosis are the third and fourth standardised moment, respectively (Casella & Berger, 2021). As the name suggests, skew describes how much the data is skewed about the mean. The kurtosis measures the tailed-ness of the distribution. A spike distribution has a kurtosis of 0, and a standard Gaussian has a kurtosis of 3. For this reason, the kurtosis values reported here are reduced by 3, which is sometimes named the*excess kurtosis*. For both measures, the ideal result is thus 0, which suggests a symmetric distribution, with tails as slim as those of a Gaussian.

**Table 3.** Moments of z-scores (i.e., the subject level deviations from the normative models), average of 10 folds.

	Skew					Excess kurtosis				
		HBR					HBR			
Feature	W-BLR	N	$S_{o}$	$S_{b1}$	$S_{b2}$	W-BLR	N	$S_{o}$	$S_{b1}$	$S_{b2}$
RCWM	**0.06**	1.34	0.14	0.14	0.14	0.57	6.05	**0.50**	**0.50**	**0.50**
RLV	0.32	2.74	**0.17**	**0.17**	**0.13**	0.89	26.39	1.40	1.08	**0.56**
WMH	0.34	6.39	**0.33**	0.34	**0.33**	**0.50**	79.03	1.54	1.52	1.39
ETICV	-0.04	0.04	**-0.01**	**-0.01**	**-0.01**	0.71	1.34	0.32	0.32	**0.30**
RIPJ	0.03	0.29	**0.0**	**0.0**	**0.0**	0.30	1.17	**0.12**	**0.12**	**0.12**
BS	**-0.0**	0.31	0.02	0.02	0.03	0.52	0.91	**0.25**	**0.25**	**0.24**
logWMH	**0.04**	0.89	0.05	0.05	0.06	**0.16**	2.07	**0.16**	**0.16**	**0.12**

Smaller is better. Note that the most direct comparison is between W-BLR,$S_{o}$and$S_{b1}$. The bold figures on the left of the vertical line show the best performing method among these methods. The$S_{b2}$models are more flexible by construction and have an additional random effect, and phenotypes that are bold on the right side of the line are those that benefit from this additional flexibilty. “Hard” phenotypes: RCWM, RLV, (log)WMH. “Easy” phenotypes: ETICV, RIPJ, BS. Abbreviations: RCWM: Right-Cerebellum-White Matter; RLV: Right-Lateral-Ventricle; WMH: White-Matter-Hypointensities; ETICV: Estimated Total Intracranial Volume; RIPJ: interim prim-Jensen thickness (right Sulcus); BS: Brain Stem; log WMH: logWhite-Matter-Hypointensities. See alsoSection 2.2.

The best results—results that are closest to 0—are printed in bold font but note that the most direct comparison is between W-BLR,$S_{o}$and$S_{b1}$. The bold figures on the left of the vertical line that separates W-BLR,$S_{o}$and$S_{b1}$from$S_{b2}$show the best performing method amongst these methods. The$S_{b2}$models are more flexible by construction and have an additional random effect. Phenotypes (rows) that are bold on the right side of that vertical line are those that benefit from this additional flexibilty. For most phenotypes,$S_{o}$or$S_{b1}$have the best result, with a few benefiting from the additional flexibility introduced by$S_{b2}$, but the difference with other methods is sometimes small. The two moments need to be weighted differently as well. For instance, for the Brain-Stem phenotype, the W-BLR has only a marginally smaller skew than$S_{o}$, but$S_{o}$,$S_{b1}$and$S_{b2}$have a significantly smaller kurtosis. It is important to remember that the kurtosis is a sum of fourth powers, and the skew is a sum of third powers. Deviations thus generally have a larger effect on the kurtosis than on the skew. For the WM-hypointensities phenotype, the W-BLR method performs on par with theSmodels regarding the skew, and better when regarding the kurtosis, indicating a better fit on those phenotypes that are also most difficult to model. The W-BLR has inherent batch effects in all aspects of the predicted percentiles, possibly explaining why it fits better to these hard phenotypes. This is further discussed inSection 3.2.1. We emphasise that the comparison is not completely straightforward with the HBR models being more conservative, as described in detail inSection 3.2.1. Despite that, the numbers indicate a fit that is overall better than W-BLR.

**Fig. 5.**
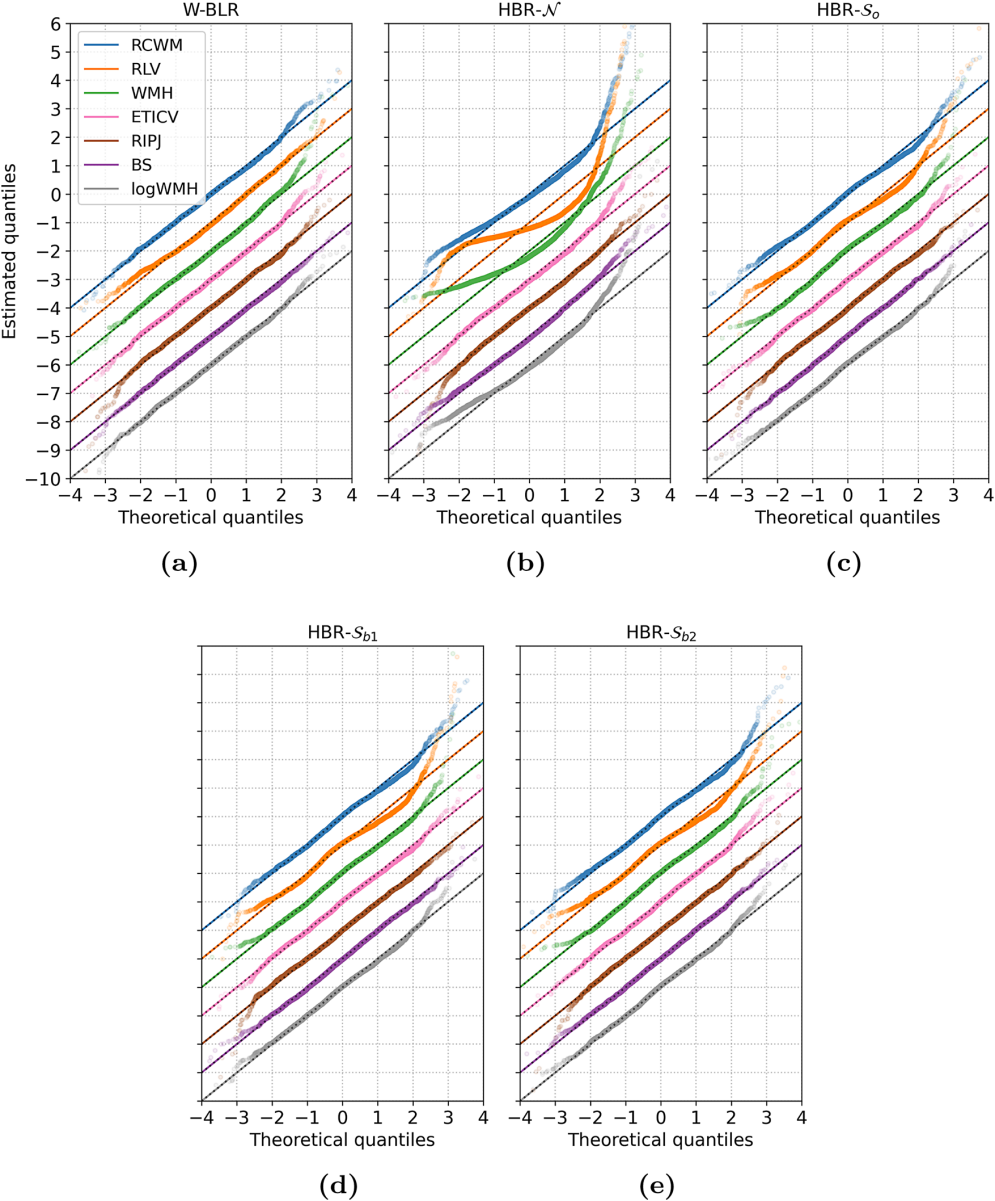
Qqplots of the estimated z-scores (i.e., the subject level deviations from the normative models) of the first test fold. Estimated z-scores of subsequent phenotypes are offset by -1 for easy comparison. For the HBR models (b), (c), (d), (e), the z-scores are computed by MAP estimation. (a) shows very straight lines, almost perfectly covering the model line. (b) shows the fit of the HBR model with Gaussian likelihood. Those phenotypes of which the residuals follow a Gaussian distribution are modelled quite well, but the limitations become very apparent when looking at the top three qqplots, which represent “hard” phenotypes. The lines are very curved, and do not reflect a good fit at all. (c), (d), and (e) show a much improved fit over (b), with the plotted points laying mostly on top of the model line. Subtle differences can be found between (d) and (e), mostly in the outer centiles, where|theoretical quantiles|>2. Abbreviations in the legend and the distinction between “hard” and “easy” phenotypes are specified inSection 2.2.

Figure 5further illustrates the good performance of the$S_{o}$and$S_{b}$models relative to theNmodel. The three hard phenotypes are not modelled well at all by theNmodel. Overall, the$S_{o}$models perform comparable to W-BLR, except for Right-Lateral-Ventricle, where the W-BLR line looks slightly better. Note that this figure shows only a random selected cross-validation fold, whereas the statistics inTable 3are averaged across all folds. Note also that the log transform does not by itself adequately model non-Gaussianiy in the WM-hypointensities phenotype, indicating that while it addresses non-negativity, it is still necessary to combine this transformation with a principled approach for modelling non-Gaussianity, such as afforded by the SHASH distribution.

**Fig. 6.**
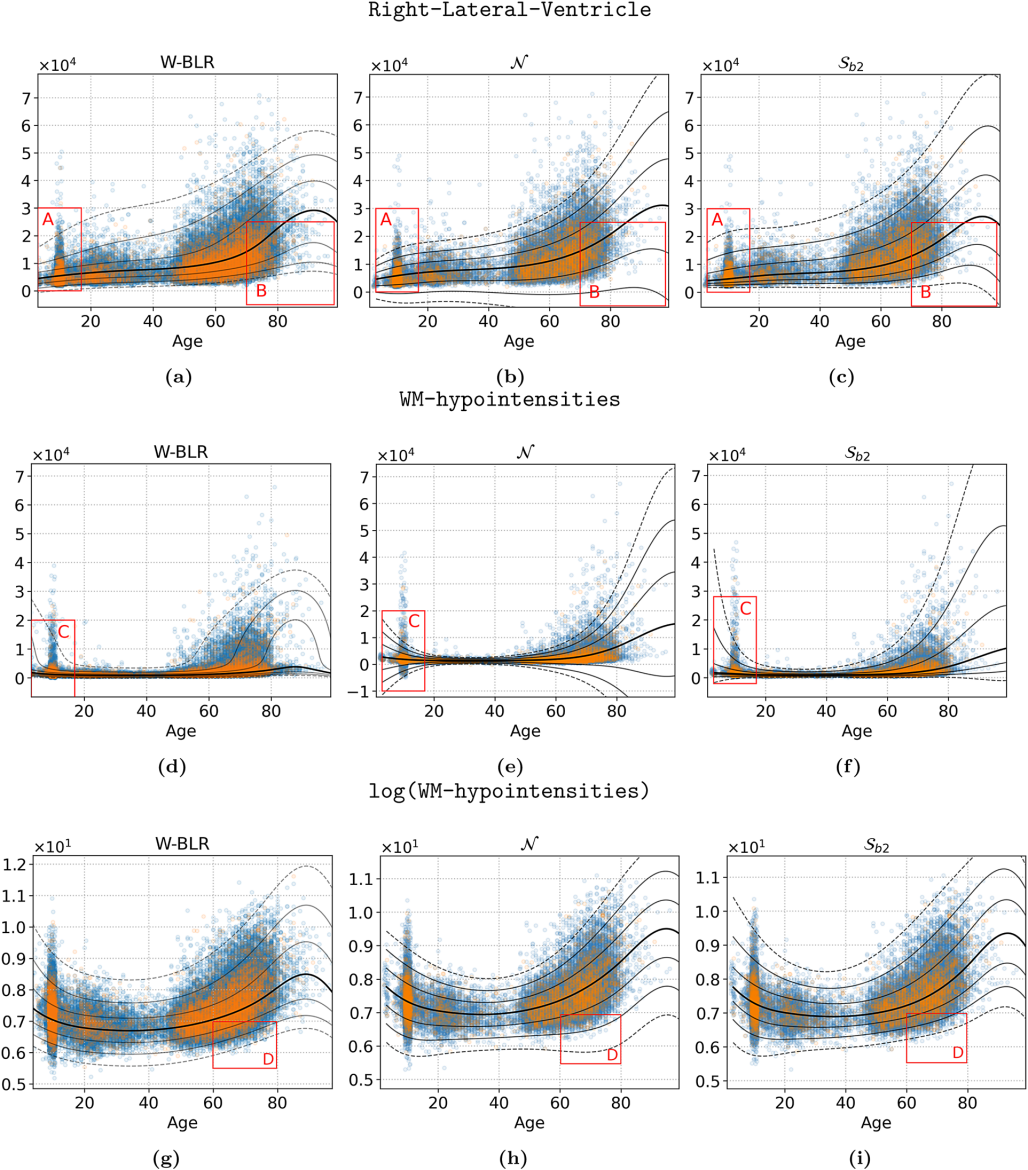
MCMC-estimated fitted centiles of Right-Lateral-Ventricle (top row (a-c)), and WM-hypointensities (middle row (d-f)), and log transformed WM-hypointensities (bottom row (g-i)) by three different HBR models (columns): all “hard” phenotypes. Blue markers indicate train data, the orange dots indicate the test data. The plotted data were corrected by subtracting the offset$\tau_{\mu }$, learned by the corresponding model (seeAppendix B). The lines mark the median (thick line) and the first three negative and positive standard deviations in the Gaussian space, acquired by transforming the values{−3,...,3}byEquation 10. Thus, the seven plotted fitted centile lines represent the standard deviations in the Gaussian space, mapped to the SHASH space, and correspond to the 0.1’th, 2.3’th, 15.9’th, 50’th, 84.1’th, 97.7’th, and the 99.9’th percentiles. Uppercase red letters highlight three areas where the difference between the methods is apparent (see text for details).

Figure 6visualises the centiles of the fitted HBR models on two hard phenotypes; Right-Lateral-Ventricle, WM-hypointensities, and the log-transformed WM-hypointensities. For brevity, we show only the$S_{b2}$model, but the fitted centiles for all models and phenotypes are shown in the appendix (i.e., including$S_{o}$and$S_{b1}$). Overall, this figure shows that the non-Gaussian models fit the data better. Focusing on area A, the W-BLR the$S_{b}$model fits the data well, but the fitted centiles of the Gaussian model (b) cannot accurately model the centiles in the lower range, and many extend implausibly into the negative range (also for e). Focusing on area B, and realising that this phenotype is a volume, and can therefore never be negative, the problem is obvious. Focusing on area C, we see that the W-BLR model must severely warp the input space to be able to accurately model the data at this point. For models (d) and (f), the 0.1’th fitted centile is still estimated to run into the negative domain, towards the range of the data points. This means that the data are not modelled accurately in those regions. As noted above, this motivates the application of the log transformation to the data before fitting which enforces positivity (g-i) and avoids the fitted centiles becoming negative toward the limits of range of the input variables. However, it is still necessary to model non-Gaussianity. Focusing on area D, we see that the Gaussian model applied to the log-transformed data has a poor fit in these regions, while the other two models perform well. These visualisations indicate that the SHASH transformation fits the data better and better accommodates site effects relative to a Gaussian model.

Another important aspect of the HBR method is the way it can deal with batch effects. Unlike the W-BLR method, where one-hot encoded^6^batch vectors are appended to the design matrix (effectively treating site as a fixed effect), causing the batch effects to directly or indirectly affect the mean, variance, skew, and kurtosis, in HBR the batch effects only affect aspects of the likelihood by explicitly giving the parameter that controls that aspect a random effect. By having a batch-specific offset inμ, sampled as a deviation from a group mean, the HBR models’ predictions are ultimately indistinguishable between batches. To assess to which degree the predictions are indistinguishable between batches, we compare the Area Under the Reciever-Operator-Curve (AUC) scores (Hastie et al., 2009), which vary between 0 and 1, in a 1v1 setting. Only site was used as a batch effect in our models, so the batch effects correspond exactly to the site-ids. For each combination of two sites, we compute the AUC score using the sklearn.metrics.roc_auc_score method, where we use the binary class labels as y_true, and the z-score predictions for y_pred. Because an AUC score of 0.5 would indicate perfect indistinguishibility, we plot the absolute deviation from the 0.5 mark inFigure 7.

**Fig. 7.**
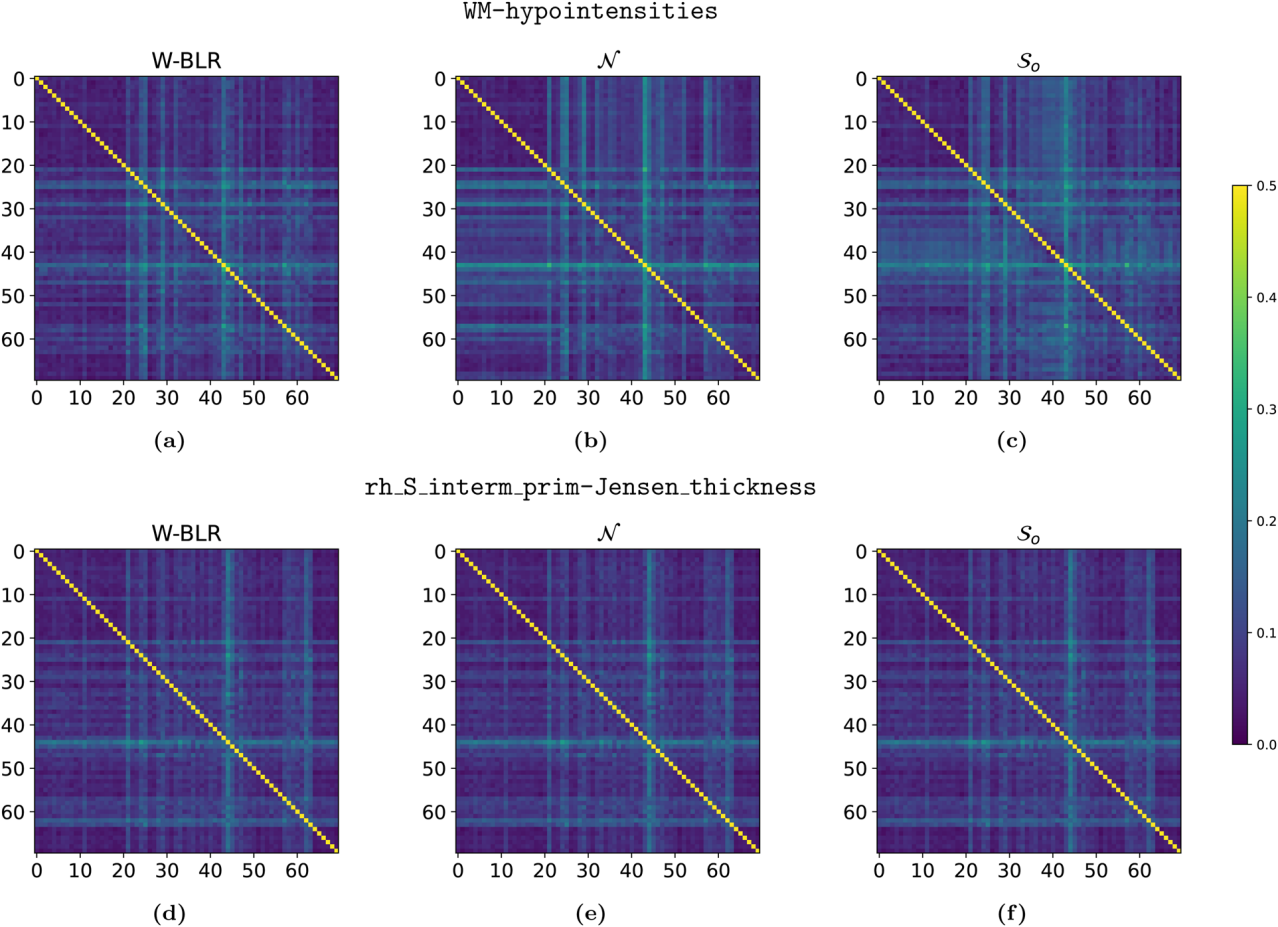
AUC scores of 1v1 site classification based on z-scores, averaged over 10 folds. The color of the x-y’th pixel represents|a−0.5|, whereais the AUC of the binary classification problem of site x and y. Thus, the diagonal is 0.5 everywhere, which provides an upper bound to the accuracy of classification for all the for the other pixels. For off-diagonal pixels, values closer to 0 are better indicating that sites cannot be distinguished because no residual site effect remains. The top row (a-c) is on z-scores of the WM-hypointensities phenotype, the bottom row (d-f) on rh_s_interm_prim-Jensen_thickness. For all models, the off-diagonal terms are somewhat homogeneously close to 0. W-BLR on WM-hypointensities (a) seems to perform a bit better than (c) and (d), with a less distinct stripe pattern. SeeSection 3.2.1for a possible explanation. All other results are listed inAppendix J, but most look similar to the plots in the bottom row; very little difference between the models is observed.

#### 3.2.1 A subtle difference between HBR and W-BLR

The comparison between HBR and W-BLR seems to indicate the W-BLR should be preferred in some cases where the modelled response variable takes on an extreme shape. However, the W-BLR method and the HBR differ in the order in which they apply certain transformations, leading to a subtle difference.

The W-BLR method applies the sinh-arcsinh transformation after applying the affine transformation for mean and variance, and the HBR method applies the sinh-arcsinh transformation before applying the affine transformation forμandσ. Becauseμandσpotentially contain batch effects, and because in W-BLR,μinfluences the shape of the distribution (as illustrated inFig. 8), the random effects in W-BLR indirectly influence the skew and the kurtosis. The better fit of W-BLR on the Right-Lateral-Ventricle and the WM-hypointensities data inTable 3may partly be due to this flexibility. We assume that in most cases, batch effects are not expected in the variance, skew, and kurtosis as much as they are in the mean. Especially for features with a variance that is small relative to the mean, a small batch effect in the mean would not constitute a large scaling in the variance. Since the skew and kurtosis are central measures—meaning that they are computed on mean-centred data that are divided by the variance—they are not influenced by scaling or shifting transformations at all. For phenotypes derived from images from a single specific scanner may be overestimated by some constant amount, but it is less likely that the shape of the trajectory of the feature depends much on the scan location itself (although seeSection 4for further discussion about this assumption). Nonetheless, a latent correlation between the site and the measurement could be present. In contrast, the HBR method provides better control because it allows modelling batch effects optionally, but not necessarily (seeAppendix B). With W-BLR, the random effects are encoded as covariates, and may therefore influence all parameters of the likelihood. The results inTable 3should be seen in light of this subtle difference. The W-BLR model may outperform HBR on some phenotypes, but the results listed under HBR are made by more conservative models, which can be controlled better in the sense that the shape parameters in HBR are implicitly regularised under the prior, whereas the shape parameters estimated by W-BLR are effectively unconstrained and can potentially more easily overfit to the data.

**Fig. 8.**
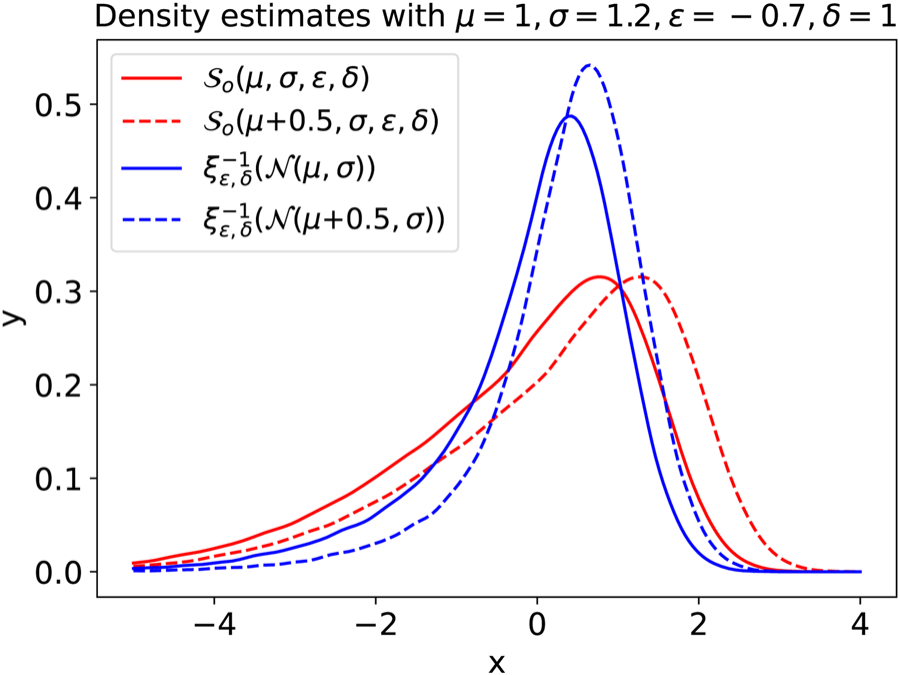
Density estimates made with 100000 samples from a standard Gaussian. For the red lines, the mean and variance (μandσ) were added after applying the sinh-arcsinh transformation to the samples. Thus, the red lines represent a SHASH*_o_*distribution. For the blue lines, the mean and variance were added to the Gaussian samples before applying the sinh-arcsinh transformation. The broken lines represent the result on exactly the same data, but with a slightly largerμ. The difference between the two red lines is limited to a shift to the right, just like one would expect when increasingμ. The difference between the blue lines is more than just a shift to the right, the broken blue line has a larger mode, and the general shape of the line has also changed slightly.Table 4further illustrates this. W-BLR uses the transformation used here to create the blue line, and hence the shape of the distribution can be controlled indirectly byΦ(the design matrix, containing a basis expansion of covariates, along with the one-hot encoded batch labels in the case of W-BLR) throughμandσ, causing it to fit better to some datasets.

**Fig. 9.**
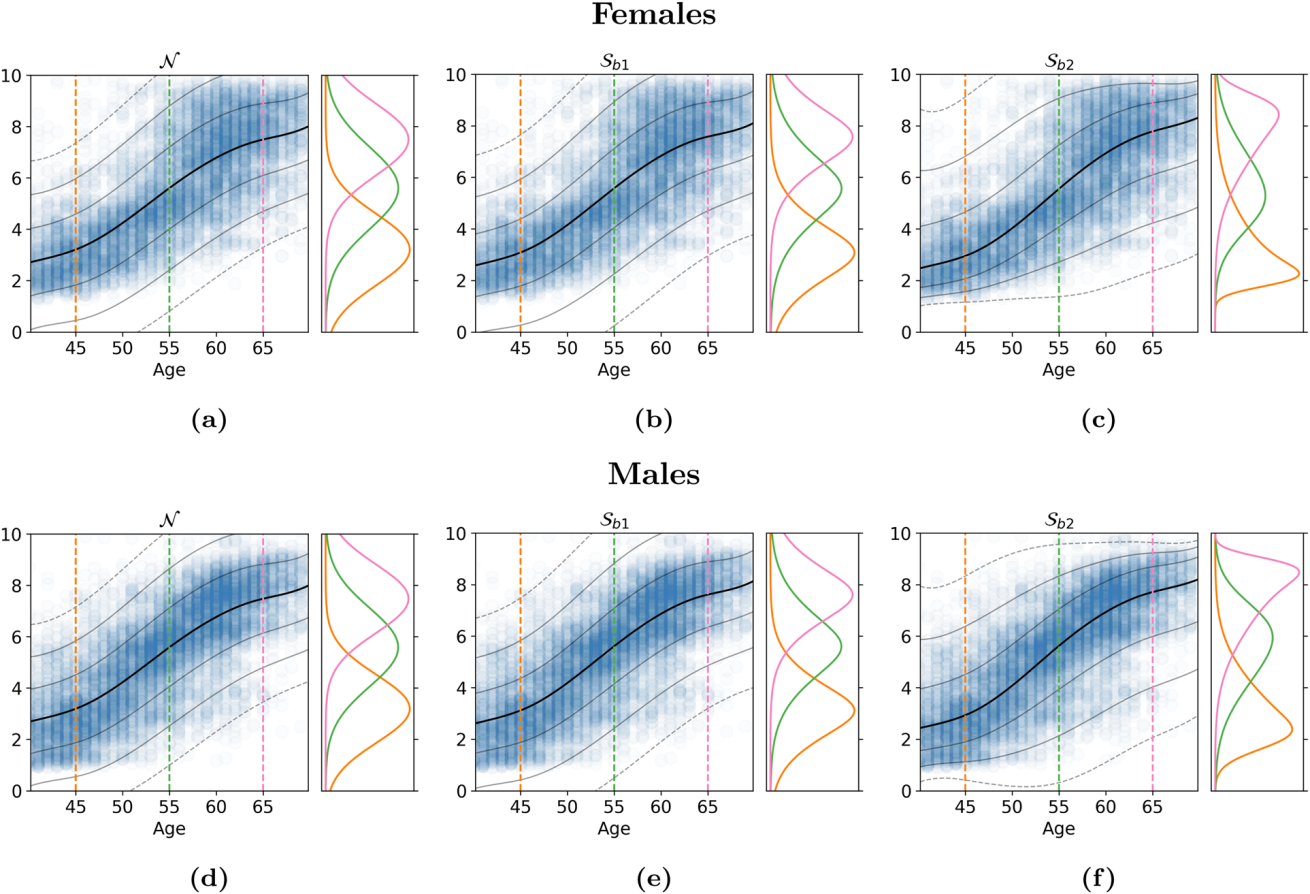
More demanding data fromZabihi et al. (2022), modelled by three different HBR models. The top row (a-c) is female data, the bottom row (d-f) is male data. The orange, yellow, and green densities plotted on the right panels are the conditional distributions at ages 45, 55, and 65. We see that although the middle models can model for skewness and kurtosis, there is barely any difference from the Gaussian likelihoods on the left. Because the data contain roughly equal amounts of positive skew and negative skew, the best bet for a point estimate is right in the middle, at zero skew. This is reflected in the conditionals, which reflect an almost perfectly Gaussian posterior. For the models on the right, which have more flexibility in the skew and kurtosis, the conditionals change shape as they move throughout the age spectrum, truly following the data distribution.

**Table 4.** Moments of the samples used inFigure 8.

	Mean	Variance	Skew	Kurtosis
$S_{o}(\mu ,\sigma ,\in ,\delta )$	-0.24	2.4	-0.96	0.9
$S_{o}(\mu +0.5,\sigma ,\in ,\delta )$	0.26	2.4	-0.96	0.9
$\xi_{\in ,\delta }^{-1}(N(\mu ,\sigma ))$	-0.05	1.27	-1.34	2.76
$\xi_{\in ,\delta }^{-1}(N(\mu +0.5,\sigma ))$	0.37	0.91	-1.31	3.37

The first two rows differ only in the mean, by the exact amount by whichμwas increased, whereas the bottom two rows differ entirely. This illustrates that in W-BLR, a change inμchanges the entire shape of the distribution.

### 3.3 Fitting highly nonlinear data with ${\text{S}}_{\text{b}}$

To demonstrate the importance of the flexibility of modelSlikelihood, we take as an example a recent study in which problematic data emerged from a dimensionality reduction (seeFig. 9).

InZabihi et al. (2022), fMRI data were compressed using an autoencoder (Kingma & Welling, 2013), and then further compressed using UMAP dimensionality reduction (McInnes et al., 2018). The result was a 2d latent representation of brain activation patterns, which followed a somewhat problematic, highly nonlinear, distribution. Positively skewed on the lower end, and negatively skewed on the higher end of the age range, this representation cannot be modelled by HBR with a Gaussian likelihood, and W-BLR is also not designed to deal with these kinds of effects. If we take$S_{b1}$, we find that we run into the limits of that model as well, because it assumes a fixed skew and kurtosis throughout the age range. With model$S_{b2}$, we have the flexibility to model heterogeneous skew and kurtosis, andFigure 9shows that this flexibility is absolutely essential for modelling this phenotype.

### 3.4 Computational complexity

We provide an indication here about the computational complexity for the time required to estimate the different variants of our models. Specifically, for the lifespan normative models presented inSection 2.2above the mean [stdev] time averaged over 10 folds and is specified as hours:minutes. This measures the time required to draw 1000 samples after 500 samples burn in was:N= 2:19 [0:36],$S_{o}$= 6:29 [1:00],$S_{b1}$= 6:54 [0:31] and$S_{b2}$= 27:26 [1:27]. Another important question in the use of MCMC samplers is determining the number of samples to acquire (i.e., the chain length). We provide a simple evaluation to determine whether 1000 samples after burn-in is sufficient. To achieve this, we compare the empirical mean and variance of different parameters across chains having 500, 1000, and 1500 samples, post burn-in. We also show Monte Carlo estimates for a representative set of parameters inFigure J.36. This shows that the parameters are effectively identical, with perhaps a slight reduction in the Monte Carlo estimates of the variance for some parameters for the short chains. Overall, this provides confidence that the chain length we employ is sufficient for reliable inferences.

## 4 Discussion

In this work, we proposed a flexible method for modelling non-Gaussianity in normative models based on the SHASH distribution. We proposed a novel reparameterisation and developed an efficient MCMC sampling approach for these models. We first applied this method to a large neuroimaging dataset and showed competitive performance with respect to competing methods, while providing full Bayesian inference and full distributions over all model parameters. Then, we applied this method to a highly challenging problem where we aim to model a set of learned parameters from a highly non-linear model (derived from an autoencoder). In this case the classical methods are not able to satisfactorily model the distribution of the brain phenotype, while the proposed method can.

Normative modelling aims to find centiles of healthy variation in neuroimaging phenotypes, much like growth charts in pediatric medicine. Many of those phenotypes follow complex nonlinear, asymmetric distributions; so for a normative model to be generally applicable, it has to be able to model features like skewness and kurtosis. Our extension builds upon the HBR framework for Normative modelling introduced by (Kia et al., 2021), which retrieves a distribution over model parameters given a prior distribution over the parameters and a likelihood over the data. The original paper reported only on results where a Gaussian likelihood was used, but the framework is flexible enough to support any parameterised likelihood. Here, we adapted the HBR framework to make use of a different one, namely the SHASH likelihood, which has additional parameters roughly modelling for skew and kurtosis.

The basic SHASH distribution inEquation 3has no parameters for location and scale, but those can be added, resulting in the SHASH*_o_*($S_{o}$) distribution, as inEquation 5. We also introduce a reparameterisation called the SHASH*_b_*($S_{b}$) distribution, which aims to remove the severe correlations by applying an affine tranformation for location and scale to a standardised SHASH distribution.

Our results on a large neuroimaging dataset show that the$S_{b}$reparameterisation in some cases converges better and more reliably than the$S_{o}$distribution. For example, in the analysis of the highly challenging data, shown inFigure 9, the$S_{o}$model did not converge. However, for most phenotypes, the convergence is similar to the original variant. A more important benefit of this reparameterisation is that the parameters of the distribution are more interpretable, as shown schematically inFigure 1and empirically inFigure 2. We note that the$S_{b}$distribution is isomorphic to$S_{o}$—the two can model exactly the same set of distributions— which might not give a preference for one distribution over the other in the context of HBR. We also observed a similar sampling performance (at least in terms of convergence) for both. However, the$S_{o}$parameterisation could in some circumstances be preferred over$S_{b}$, (e.g., for “easy” phenotypes with near-Gaussian shapes) if parameter interpretation is not of primary importance, although the latter achieved slightly better performance in our experiments. However, as noted above, this$S_{o}$model did not converge for the most difficult nonlinear phenotype we considered, in which case$S_{b}$is preferred.

We showed that HBR with an$S_{b}$likelihood performs equivalently or in some cases slightly better than Warped Bayesian Linear Regression (W-BLR) (Fraza et al., 2021) on most datasets, in terms of Gaussianity of the predicted deviation scores, although W-BLR predictions are better on specific pathological phenotypes. We argue that the W-BLR method fits better to that data due to the fact that batch effects are effectively encoded as a fixed effect and can therefore potentially affect all parameters of the likelihood, which is not always desired (seeFig. 8). The W-BLR method has no option to disable this flexibility, as it is inherent to the method. This is why we argue that the HBR method should be preferred as it supports enabling or disabling random effects in each parameter of the likelihood as a modelling choice. Moreover, the W-BLR method is unable to model the most complex phenotype we evaluated (i.e., derived from the latent representation of an autoencoder) because it does not provide the ability to sufficiently control the skewness and kurtosis across the range of the covariates. In other words, HBR provides more flexible parametric control over the shape of the distributions that the approach is able to model.

We have shown that HBR with$S_{b}$likelihood is able to model highly non-Gaussian distributions with intricate trajectories in several parameters in a way that is impossible to replicate with existing techniques. This indicates that HBR will possibly play an important role in pushing the field of Normative modelling forward.

The computational complexity of the likelihood may be the most important limitation, especially for the$S_{b2}$variant. It must be noted that the computation time is significantly reduced if fewer batch effects are present. We hope that future releases of software packages like pymc will help reduce the required computational time.

Lastly, all our work is freely available to be used and extended, in the open-source pcntoolkit package.

### 4.1 Future work

#### 4.1.1 Transfer learning

In the current work, we have not applied the method in a transfer learning setting, but the methods for doing so are described inKia et al. (2021)and the updates we provide to the pcntoolkit package support this. For transferring a learned model to a new site, one would have to create a factorised approximation of the posterior by using the MCMC samples retrieved earlier. Restricting ourselves to a known distributional form, this can easily be done by standard optimisation techniques on the log likelihood. The factorised posterior is then fixed, and can be used as an informed prior for MCMC sampling with data from a new site. The samples retrieved in this manner can then be used for approximation of z-scores or any other downstream analysis.

#### 4.1.2 Variational inference

We have shown the value of the$S_{o}$and$S_{b}$likelihoods in the context of MCMC sampling. The reparameterisation removes much of the correlation in the posterior that is problematic for sampling methods, leading to more stable results. Nonetheless, the MCMC sampling procedure is still very computationally demanding, especially on large datasets with many batch effects. A variational approach (Blei et al., 2017) may now be a viable solution to this problem. In the HBR context, the$S_{b}$reparameterisation may be particularly beneficial in this regard because it removes dependencies between the parameters, such that the posterior can be approximated by a factorised distribution. Variational inference is a fast approximation, and MCMC is a slow but (asymptotically) exact method. We believe that in some cases a fast approximation suffices, so variational approximation could be a suitable extension to the present work.

#### 4.1.3 Generalisations to more expressive noise distributions

The extension presented here is just the tip of the iceberg of Hierarchical Bayesian modelling. This extension was developed for modelling skewness and kurtosis, and while we showed that this can fit a wide range of distributions, the flexibility of the SHASH distribution is still limited. Specifically, the limitations in tail thickness are easily read from plot1d. Mixture models could further help increase the expressive power of the HBR method. One could design a mixture of a single SHASH*_b_*(or simply a Gaussian, which might be easier to fit) and two Gamma distributions, one left-tailed and one right-tailed, conveniently located as to act as surrogate tails. The centralScomponent would be dedicated to learning the shape of the bulk of the data, while the Gamma distributions absorb the largest outliers. With the current version of the pcntoolkit, this extension is now within reach, but we leave this for future work.

#### 4.1.4 Batch effects

Multidimensional batch effects are currently implemented as aDdimensional matrix in the pcntoolkit, containing one value per combination of batch effects. The number of values grows exponentially with the number of batch effects. An alternative approach is to model a separate vector for each batch effect, and to learn parameters linking the vectors together. So instead of having$\theta_{i,j}∼N(\mu_{i,j},\sigma )$, one could have$\theta_{i,j}∼N(\alpha \theta_{i}^{1}+\beta \theta_{j}^{2}+\gamma \theta_{i}^{1}\theta_{j}^{2})$. The number of required linking parameters grows as$n(n+1)/2\in O(n^{2})$, much better than the current$O(d^{n})$, wheredis the number of unique values for a batch effect. We expect that the correlation between batch effects is not sufficient to justify the approach, and we believe the most important effects in the batches can be modelled this way. In addition, throughout this work we have assumed that the batch effects are principally evident in the mean, and observed empirically that this provided reasonable performance. It is conceivable, however, that batch effects could also be evident in the variance. While the original Gaussian HBR method can easily handle batch effects in the variance, the use of the SHASH distribution complicates this somewhat; while we could add batch effects to the variance as well, this might interact with the shape and/or heteroskedasticity of the resulting model. We therefore leave this for future work.

## 5 Conclusion

We have extended a method for federated normative modelling of neuroimaging data to support non-Gaussian attributes such as skew and kurtosis. To this end, we have introduced a 4-parameter reparameterisation of the SHASH distribution, along with an accompanying software implementation. We have demonstrated that our method performs equivalently or in some cases slightly better than a baseline method on simple and complex data, thereby showing that this method can push the field of normative modelling forward.

## Figures and Tables

**Table B.5.** modelling nomenclature.

Variable	Interpretation
N∈ℕ	The number of observations, subjects, rows in the table
d	The number of clinical covariates
$X\in {\mathbb{R}}^{N\times d}$	Clinical covariates
$Y\in {\mathbb{R}}^{N\times 1}$	Response variable
B	The number of unique combinations of batch effects. So if we model 2 sexes and 7 sites, this will be 2×7=14
$Z\in {\mathbb{N}}^{N\times 1}$	A list of N indices, corresponding to the combination of batch effects that apply to the data. So every $z_{n}\in \left\{1,...,B\right\}$ . Not to be confused with the z -score.
D	The dimensionality of the basis expansion
$\Phi \in {\mathbb{R}}^{N\times dD}$	A design matrix derived from X . This can be a polynomial basis expansion, a b-spline basis expansion, or simply X .

**Fig. B.10.**
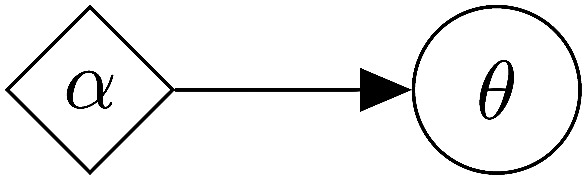
Graphical model for${ℳ}_{1}$.

**Fig. B.11.**
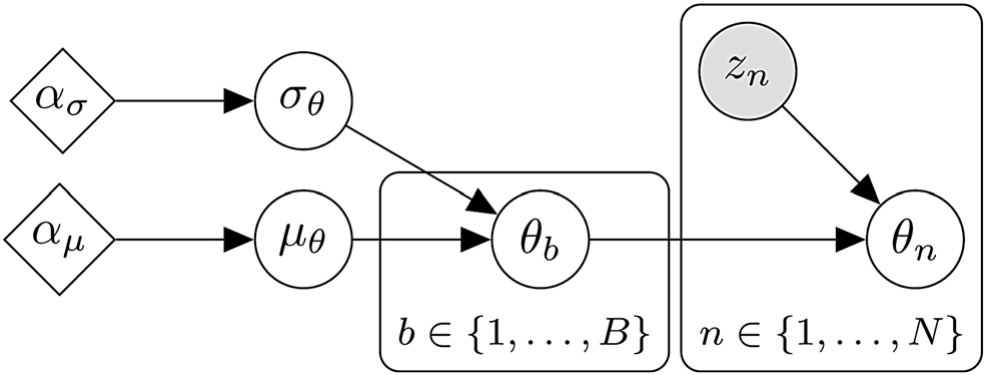
Graphical model for${ℳ}_{2a}$.

**Fig. B.12.**
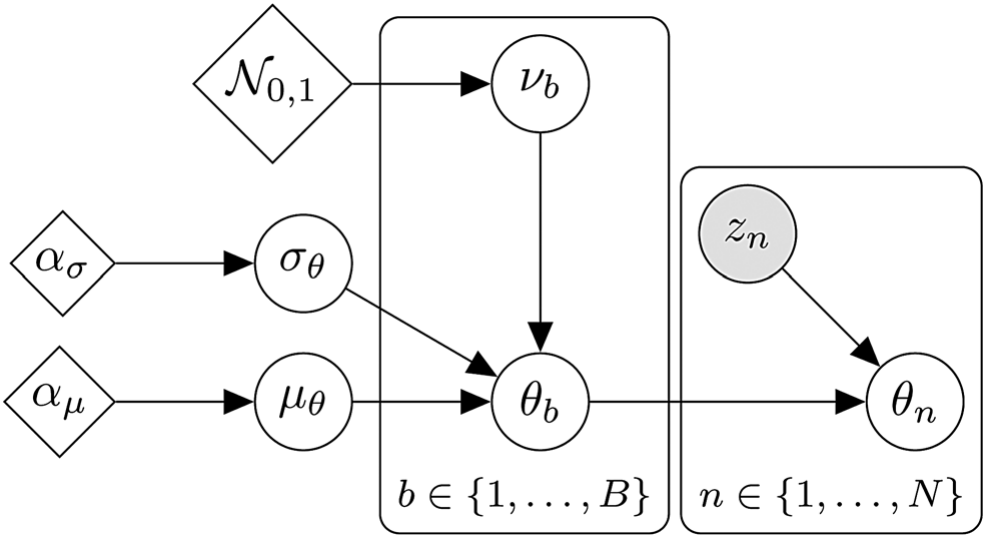
Graphical model for${ℳ}_{2b}$.

**Fig. B.13.**
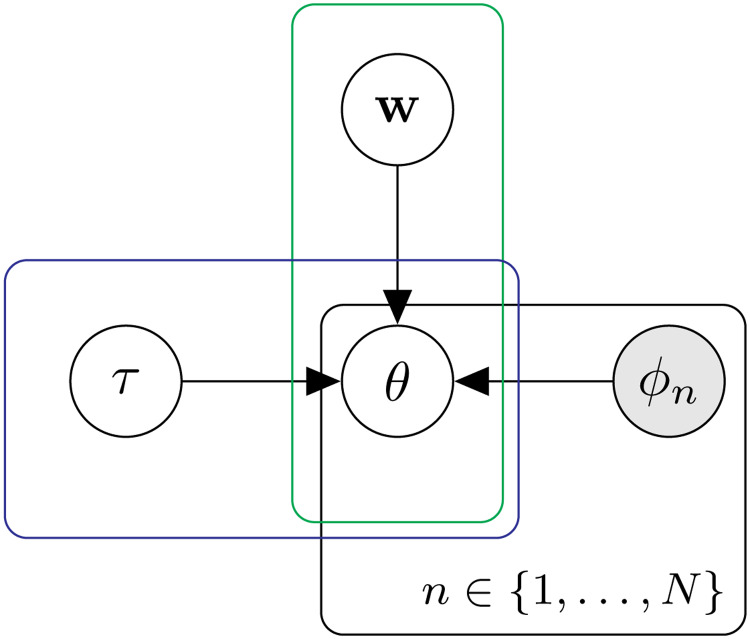
Graphical model for${ℳ}_{3.\text{*}.\text{*}}$. The green and blue box are added to emphasise thatwandτmay be modelled with batch effects, resulting in multiple values forθ.

**Table F.6.** Control data, by site.

Site	# Females	# Males	Mean age
ABCD_01	199	189	9.89
ABCD_02	252	290	10.07
ABCD_03	268	301	9.88
ABCD_04	307	324	9.81
ABCD_05	178	167	9.89
ABCD_06	286	278	9.94
ABCD_07	153	172	9.87
ABCD_08	160	176	9.95
ABCD_09	200	207	9.96
ABCD_10	280	295	9.86
ABCD_11	206	208	9.81
ABCD_12	77	84	9.88
ABCD_13	277	278	9.81
ABCD_14	267	316	10.19
ABCD_15	178	217	9.90
ABCD_16	414	507	9.90
ABCD_17	267	290	9.81
ABCD_18	160	181	9.90
ABCD_19	272	262	10.06
ABCD_20	320	320	10.05
ABCD_21	222	269	9.91
ABIDE_GU	27	27	10.42
ABIDE_KKI	65	122	10.29
ABIDE_NYU	28	107	14.40
ABIDE_USM	3	56	22.07
ADD200_KKI	27	34	10.25
ADD200_NYU	20	18	10.21
AOMIC_1000	483	445	22.84
AOMIC_PIPO2	120	89	22.18
ATV	17	60	22.67
CIN	22	44	47.89
CMI_CBIC	73	126	11.19
CMI_RU	125	251	10.53
CMI_SI	38	68	11.01
CNP-35343.0	43	47	31.94
CNP-35426.0	11	9	29.50
COI	78	46	51.86
HCP_A_MGH	86	85	59.75
HCP_A_UCLA	71	53	53.32
HCP_A_UM	120	84	61.59
HCP_A_WU	112	66	58.83
HCP_D_MGH	109	107	13.78
HCP_D_UCLA	62	65	14.13
HCP_D_UM	85	71	13.26
HCP_D_WU	75	79	13.97
HCP_EP_BWH	2	6	23.09
HCP_EP_IU	11	14	23.90
HCP_EP_MGH	3	8	27.61
HCP_EP_McL	4	9	25.15
HKH	17	12	45.41
HRC	36	13	41.69
HUH	38	29	34.74
KCL	25	16	34.12
KTT	41	87	31.03
KUT	66	93	36.50
Oasis2	127	58	76.88
Oasis3	631	921	69.78
SWA	15	85	28.48
SWU_SLIM_ses1	318	230	20.07
UCDavis	62	74	3.12
UTO	106	96	35.19
Cam	329	318	54.19
Delta	18	31	50.44
ds001734	60	48	25.54
ds002236	38	48	11.49
ds002330	37	29	26.62
ds002345	131	76	21.69
ds002731	28	31	21.25
ds002837	42	44	26.73
hcp_ya	606	507	28.80
Ixi	313	245	48.71
Nki	308	174	42.63
Pnc	701	677	14.21
Top	134	158	34.57
ukb-11025.0	12944	12042	62.98
ukb-11027.0	5438	4534	64.39

**Table F.7.** Patient data, by site.

Site	# Females	# Males	Mean age
ABIDE_GU	8	39	10.94
ABIDE_KKI	19	58	10.22
ABIDE_NYU	16	111	12.83
ABIDE_USM	2	72	21.60
ADD200_KKI	10	12	10.21
ADD200_NYU	14	38	10.05
CIN	28	45	56.80
CNP-35343.0	30	33	35.33
CNP-35426.0	15	44	34.62
COI	40	29	45.07
HCP_EP_BWH	8	15	22.38
HCP_EP_IU	22	37	22.96
HCP_EP_MGH	3	7	20.31
HCP_EP_McL	15	16	23.56
HKH	13	20	44.81
HRC	10	6	40.50
HUH	25	32	43.33
KCL	75	29	31.44
KTT	21	26	37.89
KUT	30	31	41.70
Oasis2	82	97	76.94
Oasis3	158	123	75.37
SWA	19	115	33.61
UTO	54	95	35.61
Delta	142	160	41.25
Top	254	277	32.42

**Table F.8.** Patient data, by group.

Group	# Females	# Males	Mean age
AD	142	94	75.75
ADHD	40	71	17.60
ASD	61	389	19.08
BD	130	106	34.67
Converted	24	13	79.75
Demented	58	84	76.21
MCI	16	29	73.40
MDD	260	239	40.26
MDD_motar	30	21	35.50
Other	15	26	34.21
Pain	25	39	55.28
Patient	72	98	30.01
Patient_motar	32	38	40.20
SZ	131	225	34.85
Stroke	3	6	67.66
Otherpsychosis	32	52	29.76
Schizoaffective	23	10	35.69
Schizophreniform	11	12	25.43

**Fig. G.14.**
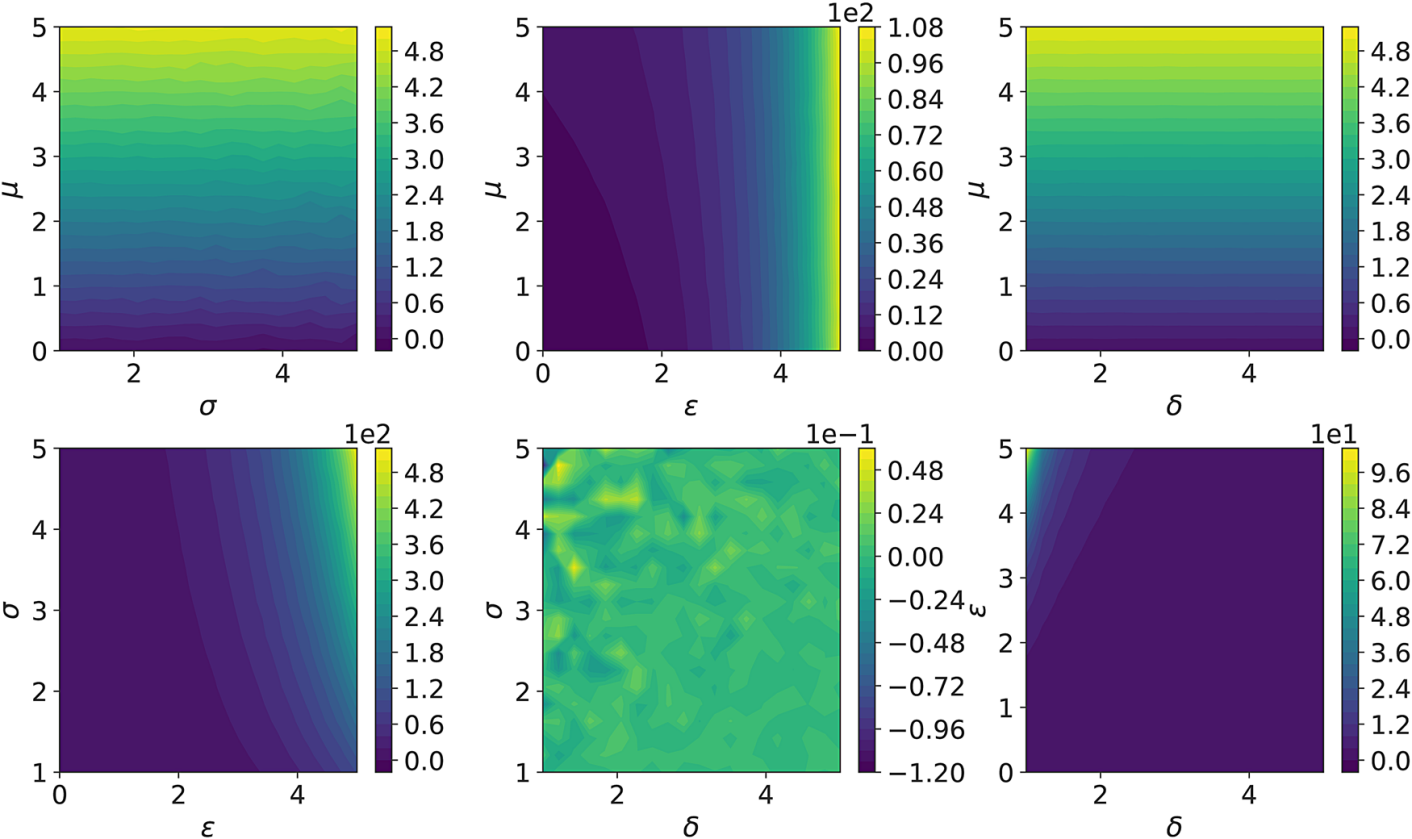
SHASH*_o_*first momentE[x].

**Fig. G.15.**
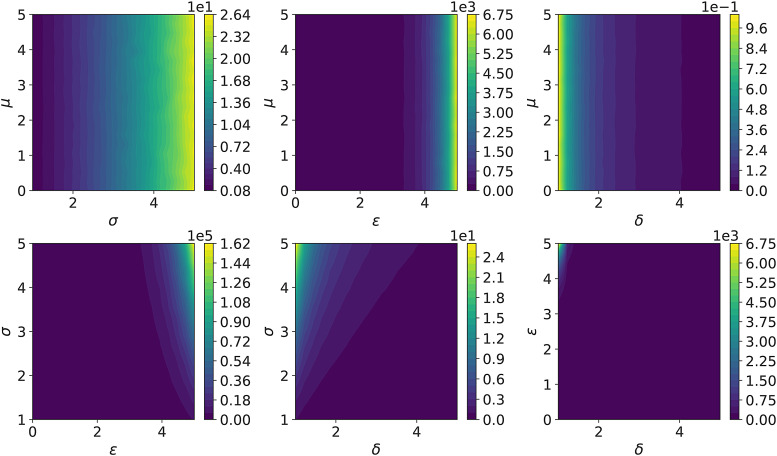
SHASH*_o_*second moment$E[{\left(x-\mu \right)}^{2}].$

**Fig. G.16.**
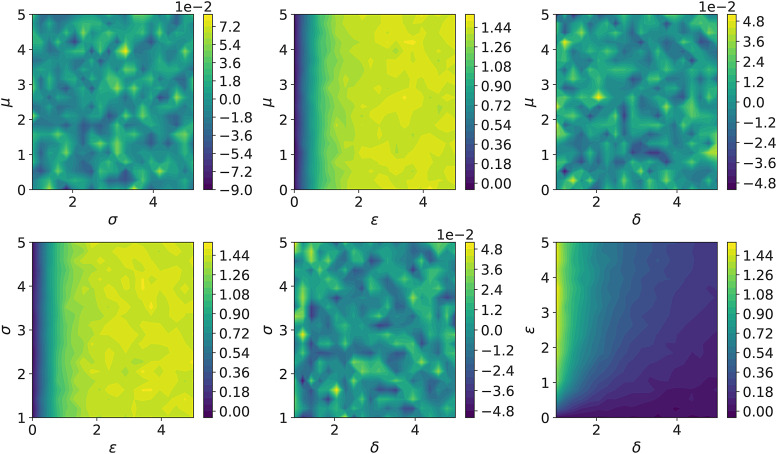
SHASH*_o_*third moment$E[{\left((x-\mu )/\sigma \right)}^{3}]$.

**Fig. G.17.**
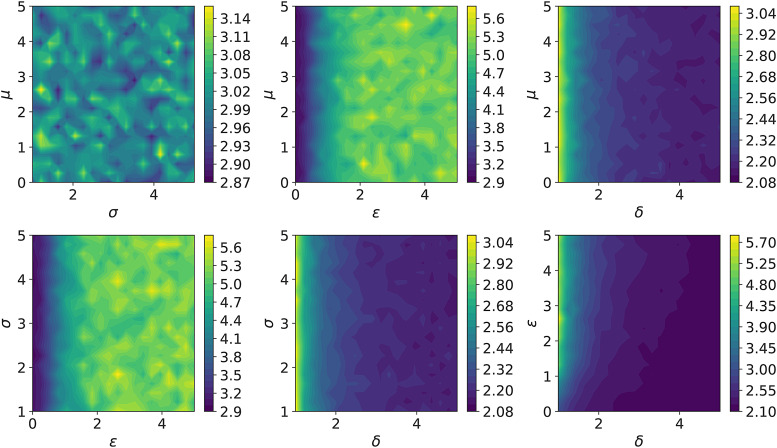
SHASH*_o_*fourth moment$E[{\left((x-\mu )/\sigma \right)}^{4}]$.

**Fig. H.18.**
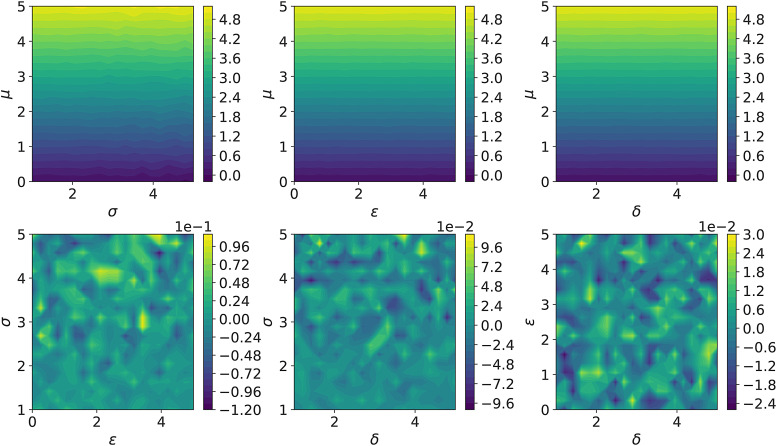
SHASH*_b_*first momentE[x].

**Fig. H.19.**
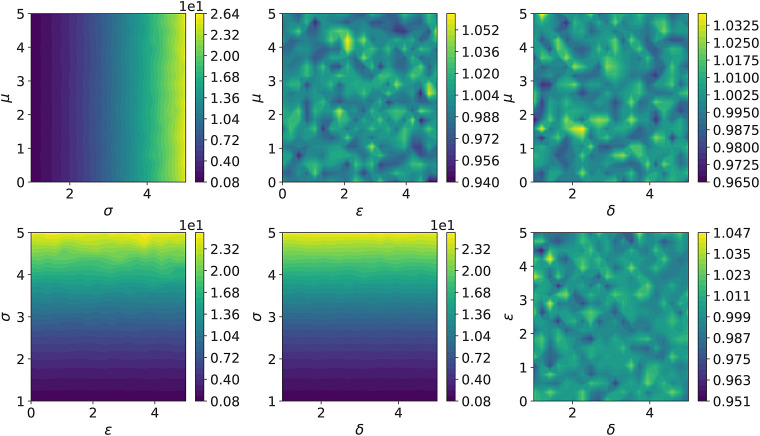
SHASH*_b_*second moment$E[{\left(x-\mu \right)}^{2}]$.

**Fig. H.20.**
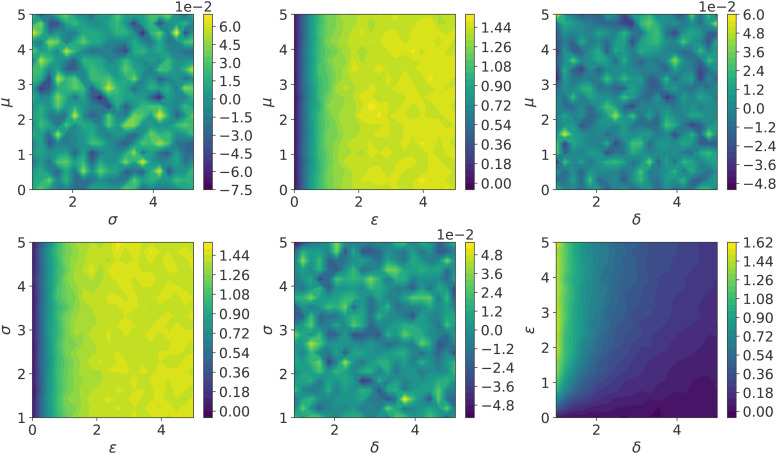
SHASH*_b_*third moment$E[{\left((x-\mu )/\sigma \right)}^{3}]$.

**Fig. H.21.**
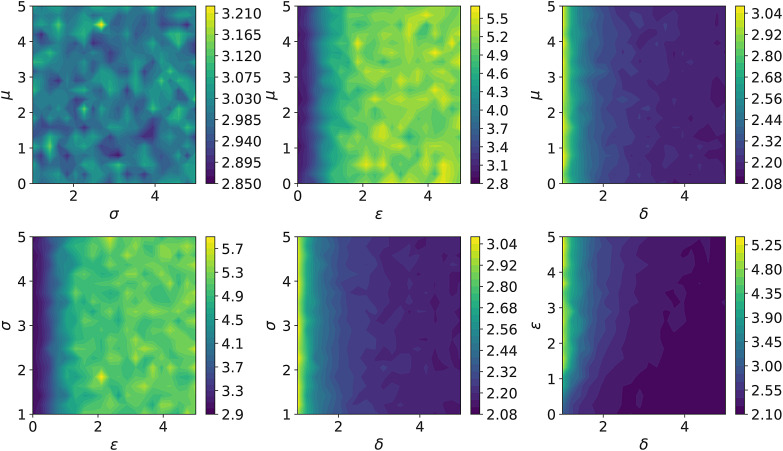
SHASH*_b_*fourth moment$E[{\left((x-\mu )/\sigma \right)}^{4}]$.

**Fig. I.22.**
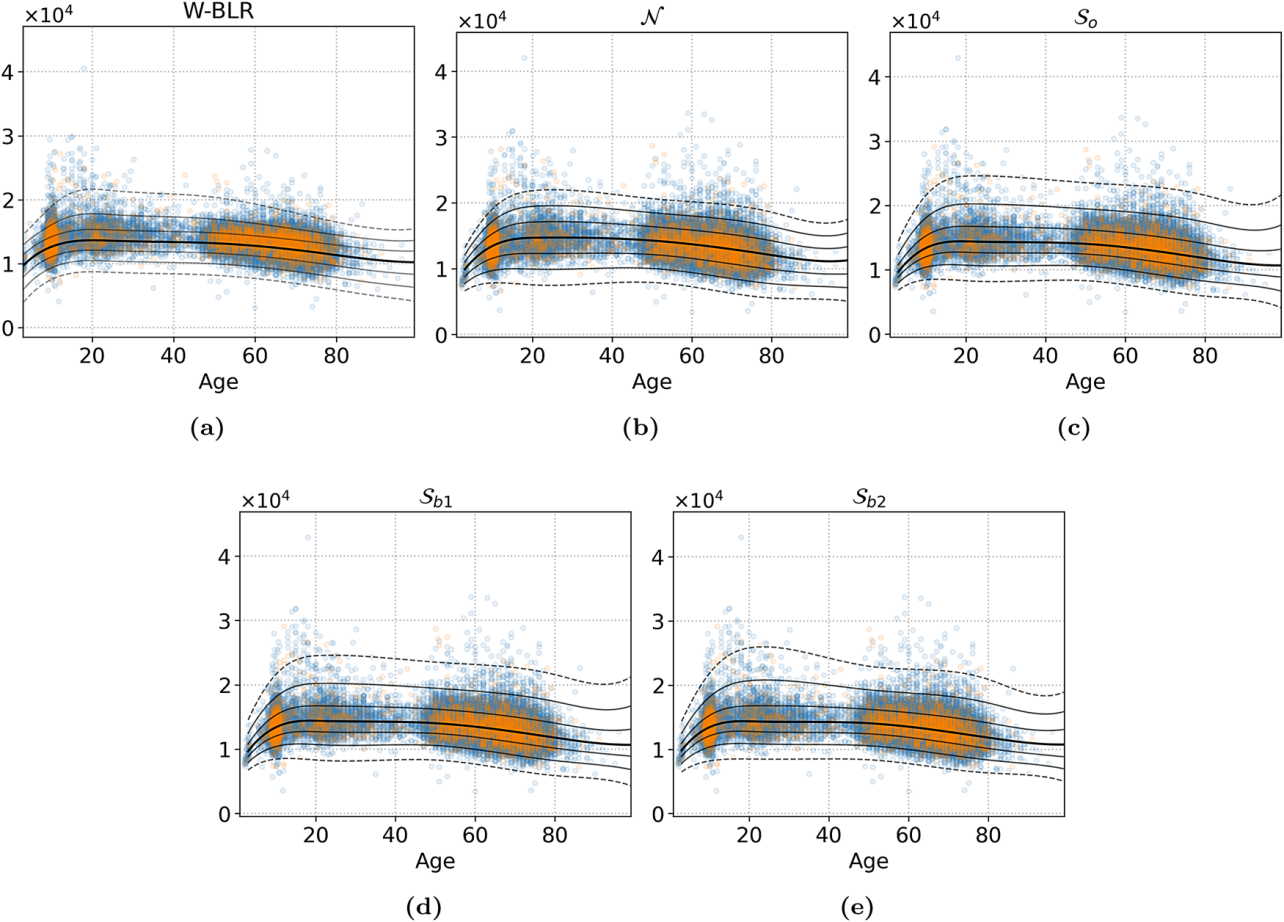
Right-Cerebellum-White-Matter.

**Fig. I.23.**
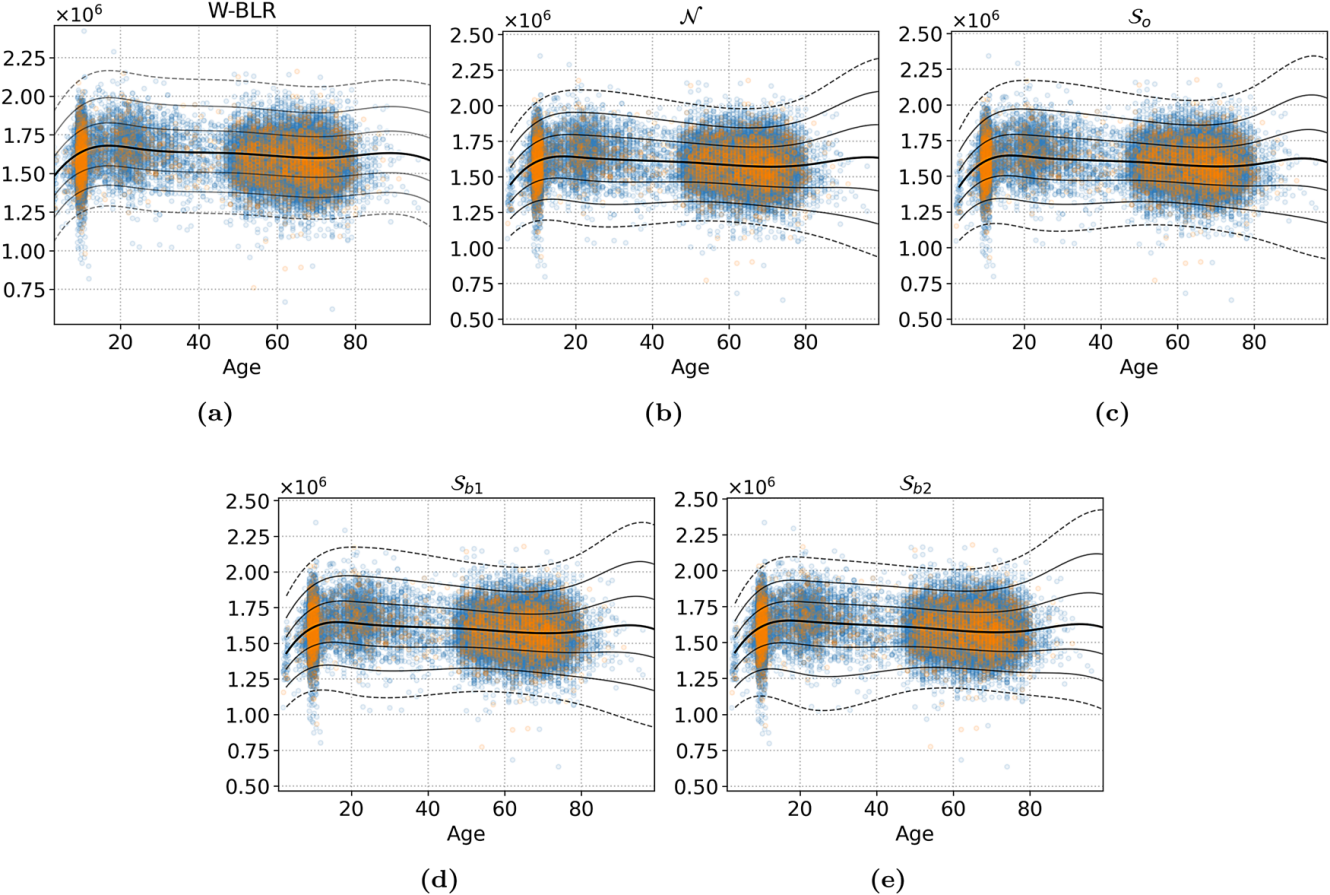
EstimatedTotalIntraCranialVol.

**Fig. I.24.**
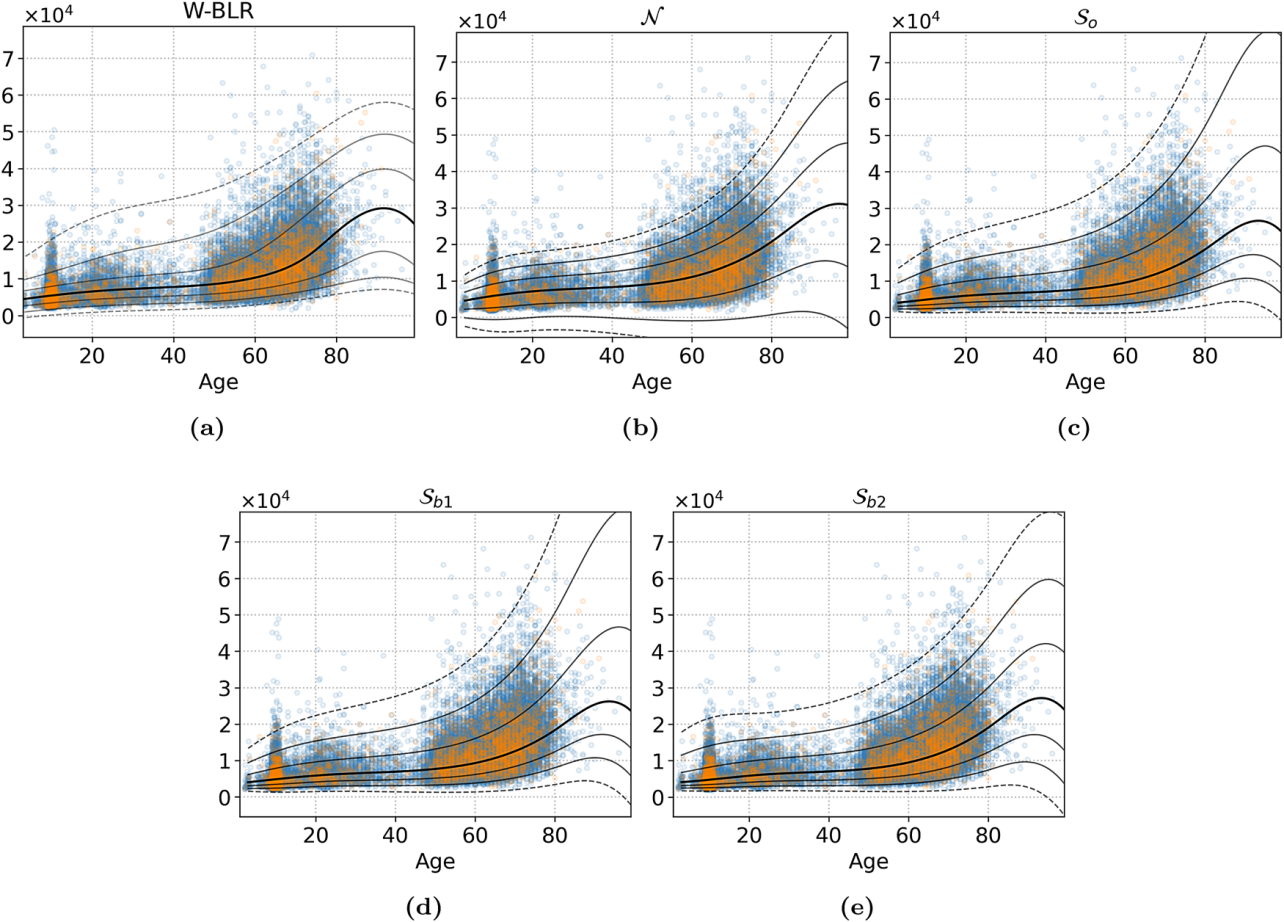
Right-Lateral-Ventricle.

**Fig. I.25.**
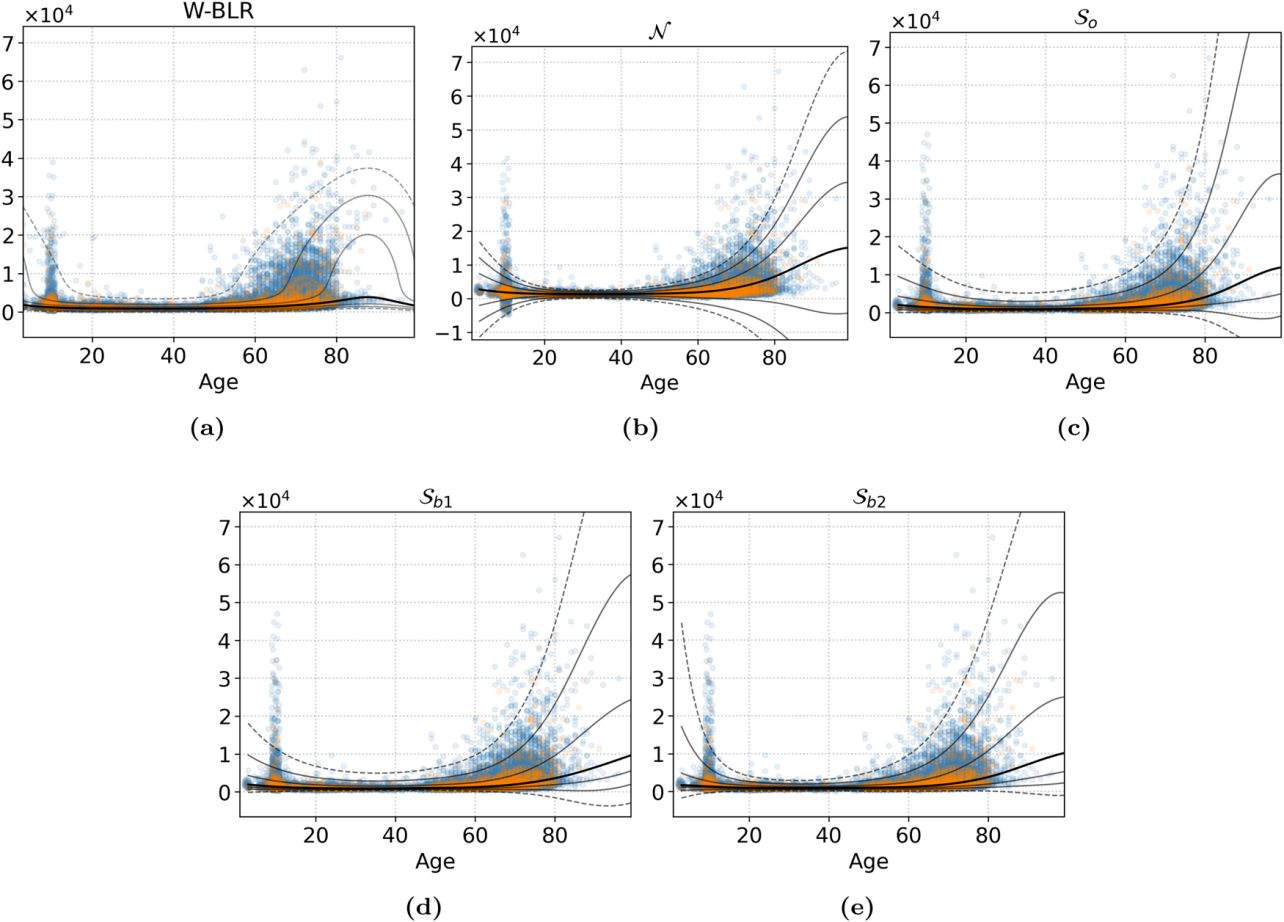
WM-hypointensities.

**Fig. I.26.**
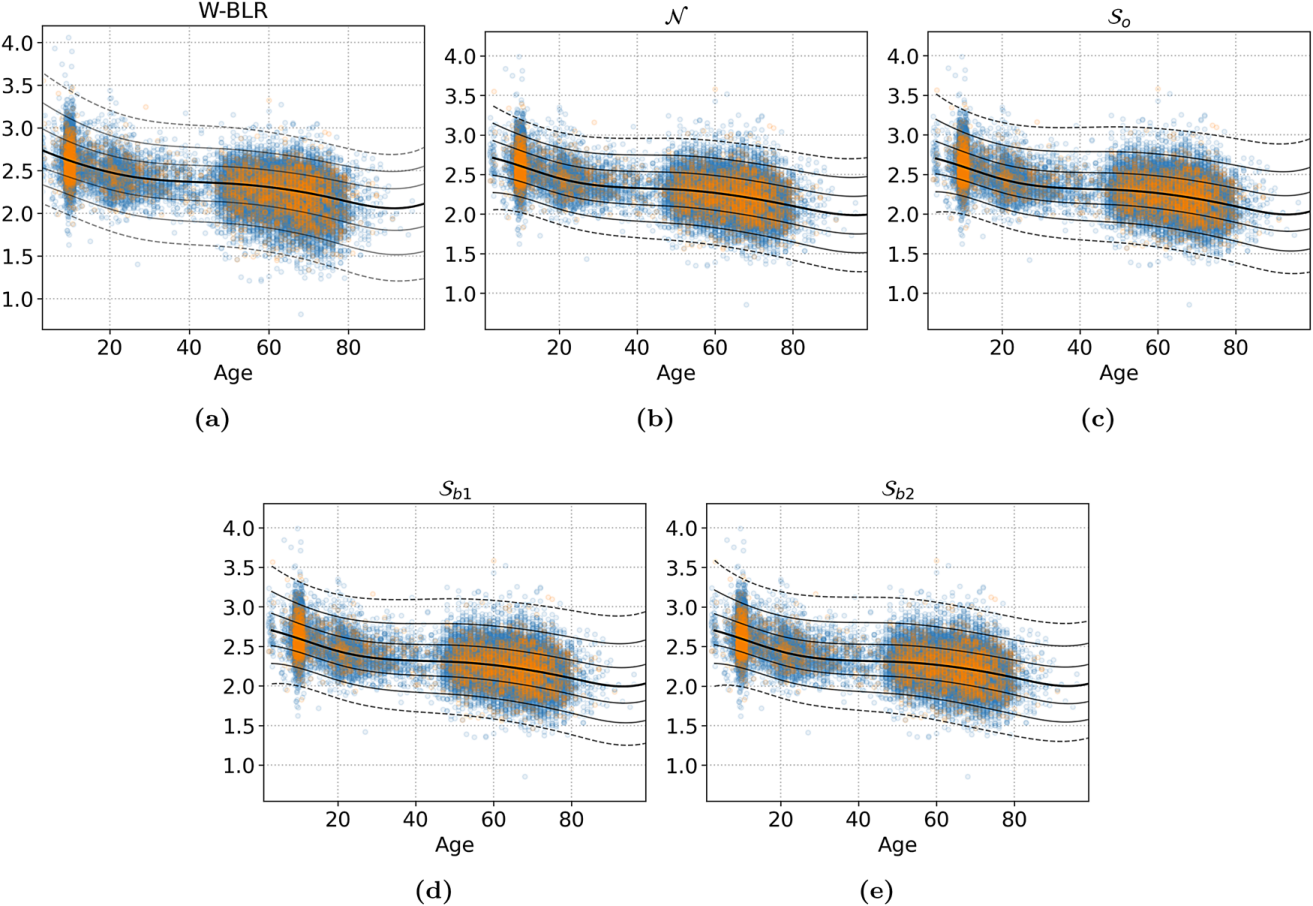
rh_S_interm_prim-Jensen_thickness.

**Fig. I.27.**
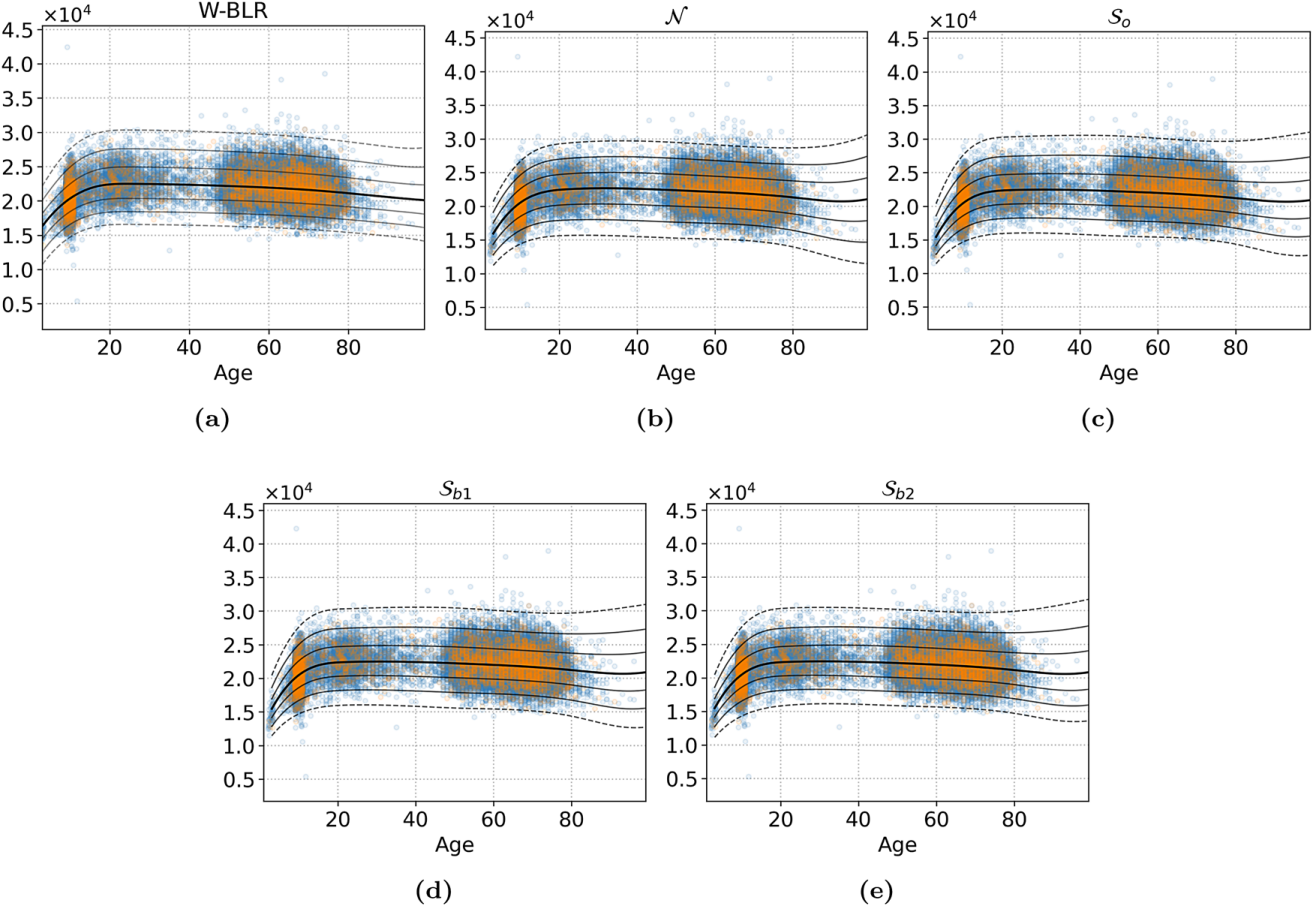
Brain-Stem.

**Fig. I.28.**
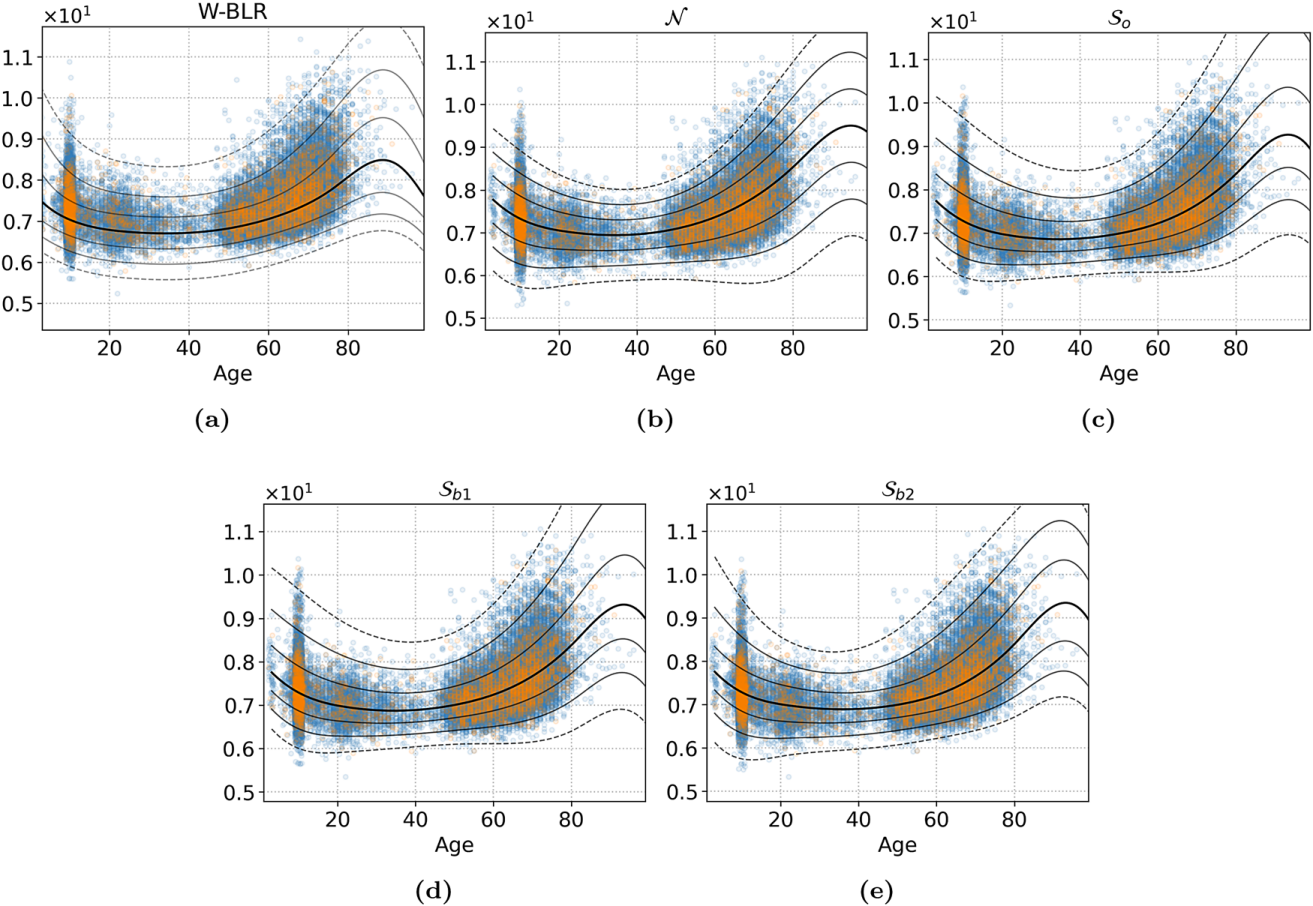
log WM-hypointensities.

**Fig. J.29.**
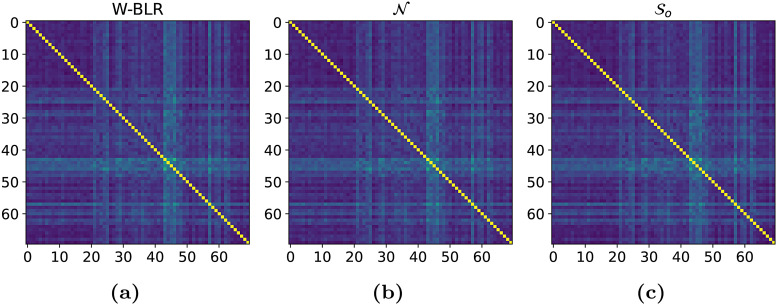
AUC scores of the Right-Cerebellum-White-Matter phenotype.

**Fig. J.30.**
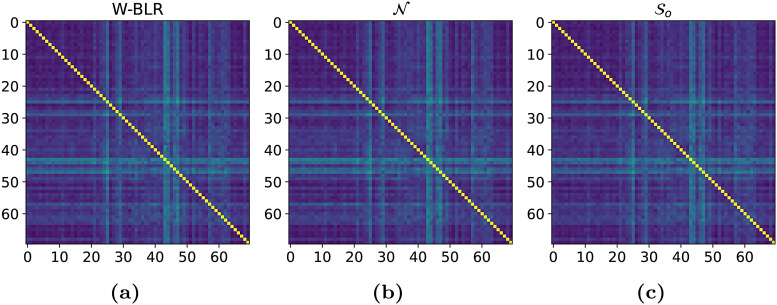
AUC scores of the Right-Lateral-Ventricle phenotype.

**Fig. J.31.**
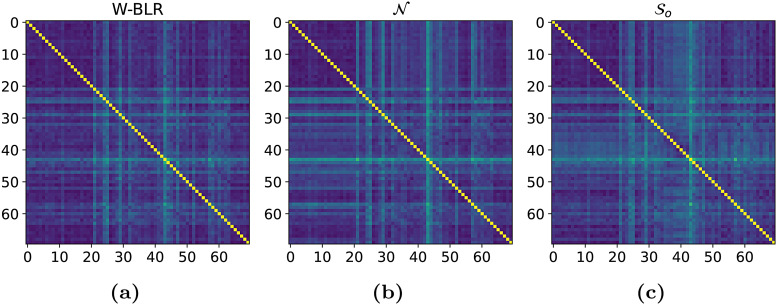
AUC scores of the WM-hypointensities phenotype.

**Fig. J.32.**
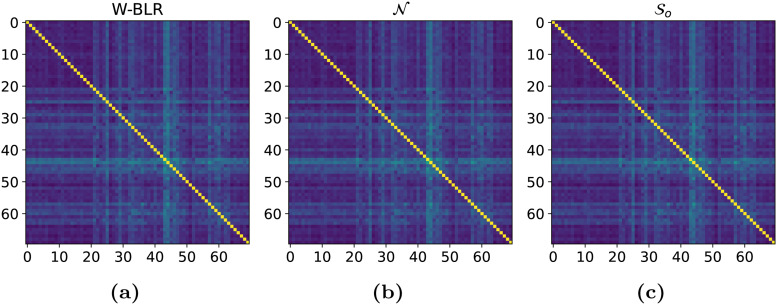
AUC scores of the EstimatedTotalIntraCranialVol phenotype.

**Fig. J.33.**
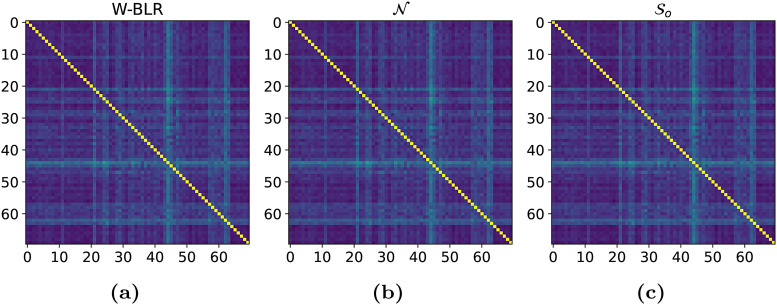
AUC scores of the rh_S_interm_prim-Jensen_thickness phenotype.

**Fig. J.34.**
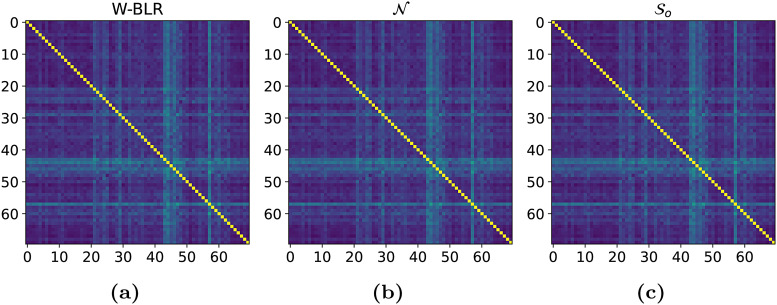
AUC scores of the Brain-Stem phenotype.

**Fig. J.35.**
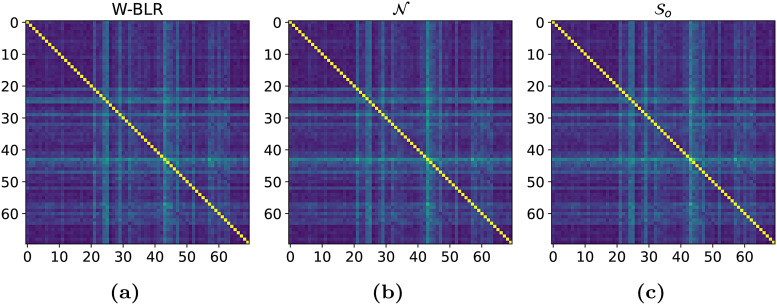
AUC scores of the Brain-Stem phenotype.

**Fig. J.36.**
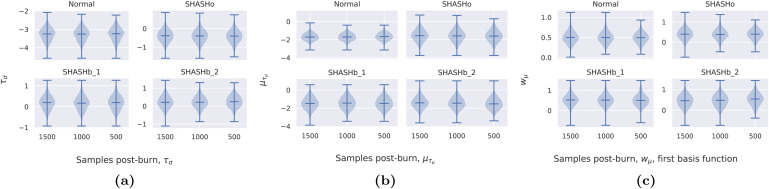
Effect of post-burn chain length on selected parameters.

## Data Availability

Most data used in this manuscript are publicly available and can be downloaded from the repositories providing the data. SeeRutherford et al. (2022)for details. Data from the “Thematically Organised Psychosis” sample is available under reasonable request by contacting the responsible PIs (L.T.W. and O.A.A.). A software implementation of the tools presented here is provided in the pcntoolkit package and the scripts for running the experiments are also provided online via GitHub.

## Footnotes

1 The transformation of the Gaussian samples Z via the sinh-arcsinh transform only relates to the construction of the SHASH distribution, and it does not relate to z-scoring the data prior to analysis. Nevertheless, we did standardize all variables used in the analysis before fitting since it can improve the performance of the samplers.

2 A differentiable function with a known and differentiable inverse.

3 We have only real inputs because y is real and the cosh and sinh^−1^functions map real numbers to real numbers.

4 Correlations like this cause problems for inference in HBR because of its reliance on MCMC sampling. In MCMC sampling, an algorithm traverses a path of pseudo-random points in parameter space, storing visited points as samples. Under the conditions that (i) the Markov chain has the posterior distribution as its stationary distribution, and (ii) detailed balance is satisfied, the samples that are retrieved follow the posterior distribution over model parameters, and can thus be used to approximate distributions. Correlations in the parameter space inhibit the sampler from efficiently exploring the parameter space, resulting in samples that do not follow the posterior very well, or do not converge to the target density at all.

5 Note that this differs slightly from the sample size in Rutherford et al. because we removed several small clinical samples for which permission for sharing was not available.

6 A one-hot encoding of an N-ary class label is a vector with a single ‘1’ at the index of the class that it labels, and zeros everywhere else.

## Appendix A. Sinh-Arcsinh Transformation

$$S_{\in ,\delta }(x)=\text{sinh(}\delta {\text{sinh}}^{-1}(x)-\in \text{)}$$
(A. 1)

$$S_{\in ,\delta }^{-1}(x)={\text{sinh((sinh}}^{-1}\left(x\right)+\in \text{)}/\delta \text{)}$$
(A. 2)

$$=\text{sinh}\left({\text{sinh}}^{-1}\left(x\right)\delta^{-1}+\in \delta^{-1}\right)$$
(A. 3)

$$=S_{\delta^{-1},\in \;\delta^{-1}}\left(x\right)$$
(A. 4)

## Appendix B. Model Specification

Every phenotype has its own defining characteristics. Some may be severely skewed, while others appear to be nicely Gaussian. We may see strong correlations between age and variance (heteroskedasticity) in one, while another seems to retain a consistent variance throughout the entire age spectrum. Batch effects like sex-specific differences, or site-specific noise characteristics may or may not be present in the data. Characteristics like these can be learned by a sufficiently flexible model. However, Occam dictates that we should prefer a simple model over a complex model, if they perform equally well. A model is therefore optimally defined if it has enough flexibility to capture the parameters of interest, whilst being constrained in every other way. An ideal modelling framework would allow specifying constraints on all aspects of the model, be flexible enough to support a wide range of models, while still remaining concise. Here, a model definition framework is proposed that allows us to concisely write down a wide range of different models. This framework will simplify the later sections of this report. In addition, the framework introduced here aligns with the implementation of the source code in the pcntoolkit software package. The graphical notation used here is slightly adapted fromBishop (2006)chapter 8, where here we indicate the range and name of the iterator variables more explicitly in the plates, i.e.,Nbecomesn∈{1,...,N}. For the remainder of this section, we will uphold the nomenclature inTable B.5. Without loss of generality, let us now focus on modelling a single parameter,θ. For more complex distributions that take more parameters, like almost any distribution, we just apply what follows to every parameter separately.

### Appendix B.1. Constant θ

Here, we discuss simple models whereθis constant for the whole spectrum ofX, save batch effects.*Model*${ℳ}_{1}$. Let${ℳ}_{1}$represent the case whereθdoes not depend onXorZ, i.e., it takes a single value for all$y_{n}$. The only dependency ofθin${ℳ}_{1}$is on the (set of) hyperparametersαthrough its prior distributionp:

$$\begin{array}{ccc} \theta ∼p_{\theta }\left(\alpha \right) & & \end{array}$$
(B. 1)

andFigure B.10shows the graphical model.

#### Appendix B.1.1 Constant θ with batch effects

Combinations of site, sex, and possible other factors define distinct groups called*batches*. Here, we use the term*batch effects*to indicate possibly unknown differences between known and distinct batches present in the data. HBR can deal with these batch effects in a principled way by modelling batch-specific parameters as deviations from a learned group mean. This allows information from all batches to flow into the hyperpriors, which, in turn, influence the estimated individual parameters for each batch. The models${ℳ}_{2a}$and${ℳ}_{2b}$encode this.

*Model*${ℳ}_{2\text{a}}$. CThe next simplest case is model${ℳ}_{2a}$, in whichθdoes depend onZbut not onX. This encodes the belief that the parameter is constant for allx, but that it may be influenced by batch effects. We sample the batch effects ($\theta_{b}$) as Gaussian deviations from a group mean ($\mu_{\theta }$) with a group variance ($\sigma_{\theta }^{2}$), such that the sites can learn from each other. Additionally, we have that under the appropriate prior, the group variance acts as a regulariser.

$$\begin{array}{ccccccc} \mu_{\theta }∼p_{\mu }(\alpha_{\mu_{\theta }}) & & & & & & \\ \sigma_{\theta }∼p_{\sigma }(\alpha_{\sigma_{\theta }}) & & & & & & \\ \theta_{b}∼N(\mu_{\theta },\sigma_{\theta }) & & b\in \left\{1,\ldots ,B\right\} & & & & \\ \theta_{n}=\theta_{z_{n}} & & n\in \left\{1,\ldots ,N\right\} & & & & \end{array}$$
(B. 2)

*Model*${ℳ}_{2b}$. The phenomenon known as the funnel is an issue that commonly afflicts hierarchical models, and it may occur in model${ℳ}_{2a}$. To give a brief explanation, when the parameter$\sigma_{\theta }$is small, but we cannot determineθwith enough confidence, the posterior distribution attains a wide support in the domain of$\sigma_{\theta }$, including very small values. If we sample in those regions where$\sigma_{\theta }$is very small, the valuesθall collapse to$\mu_{\theta }$, and sampling ofθbecomes very hard. Most samples ofθwill be rejected, since the proposal distribution is tuned for a larger step size. For a more thorough explanation, see (Betancourt & Girolami, 2015). In those situations where we fear a funnel may occur, we apply the following reparameterisation as a remedy; the batch-specific deviation from the group mean is determined by sampling the group standard deviation ($\sigma_{\theta }$) from a positive distribution, and then multiply that with an*offset*($\nu_{b}$) sampled from a central member of a symmetric distribution—like the standard Gaussian—for eachb∈{1,…,B}. Sampling like this is sometimes called a non-centred sampling approach, as opposed to the centred approach in model${ℳ}_{2a}$. Here, we propose model${ℳ}_{2b}$:

$$\begin{array}{ccccccc} \mu_{\theta }∼p_{\mu }(\alpha_{\mu_{\theta }}) & & & & & & \\ \sigma_{\theta }∼p_{\sigma }(\alpha_{\sigma_{\theta }}) & & & & & & \\ \nu_{b}∼N(0,1) & & b\in \left\{1,\ldots ,B\right\} & & & & \\ \theta_{b}=\mu_{\theta }+\sigma_{\theta }\nu_{b} & & b\in \left\{1,\ldots ,B\right\} & & & & \\ \theta_{n}=\theta_{z_{n}} & & n\in \left\{1,\ldots ,N\right\} & & & & \end{array}$$
(B. 3)

### Appendix B.2 θ as a function of X

More complex models can be defined if we letθfollow a linear relationship with the basis expansionΦ(X)through a set of weights$\text{w}\in {\mathbb{R}}^{dD}$and an interceptτ∈ℝ. We samplewandτfrom their own respective distributions, which is where the definitions fromAppendix B.1are re-used.

*Model*${ℳ}_{3.*.*}$. Sincewandτare both constant parameters with possible batch effects, we can model them exactly like we didθin${ℳ}_{1}$, or$\theta_{z_{n}}$in models${ℳ}_{2a}$and${ℳ}_{2b}$. The definitions there are general enough to allow modellingwas distributed according to a multidimensional density.

The subscript of these linear models consists of three terms, separated by periods;${ℳ}_{3.*.*}$. The first term is a 3, signifying that this is a linear model. The second and third terms indicate the model types that are used forwandτ, respectively. For instance:${ℳ}_{3.1.1}$is a model whereθis a linear function ofΦthroughwandτ, which are both constants likeθin${ℳ}_{1}$. Model${ℳ}_{3.2a.2b}$encodes for the model with a linearθ, wherewandτhave unique values for each batch effect, but the former is modelled by the centred approach as in model${ℳ}_{2b}$, and the latter by the non-centred approach explained in model${ℳ}_{2a}$. Bothwandτcan thus be modelled in three distinct ways, allowing us to define a total of nine different models with linearθin this way.θcan thus be modelled in a total of 12 ways: Three in which it is fixed, and nine in which it has a linear dependence onΦ.

τ∼likeEq. B.1,B.2, orB.3

w∼likeEq. B.1,B.2, orB.3

$$\theta =\phi_{n}^{T}\text{w}+\tau \;\;\;\;\;\;n\in \left\{1,...,N\right\}$$

### Appendix B.3 Hypothetical application

The proposed framework allows us to concisely specify how parameters are modelled.

*Scenario.*A pattern that is commonly observed in neurological phenotypes is the following: As the age of the participant (X) increases, the phenotype (Y) increases first (until around 25), and then starts to decrease. However, the variance in the phenotype increases with age as well. The data show some positive skew, and may also be slightly kurtotic. Furthermore, the data are collected from various sites, so site-effects ($Z_{1}$) are expected, and sex-related differences ($Z_{2}$) need to be accounted for as well.

*Modelling.*Clearly, the parameter that controls the mean should be a function of age, but the non-monotonic behaviour of the mean guides us away from a directly linear approach. Rather, we would use a polynomial basis expansion, or a regression spline (Boor, 1978). We usually prefer the regression spline basis over a simple polynomial basis, because it does not induce global curvature (Fraza et al., 2021). A Gaussian distribution would not be able to capture the observed skew, so the decision for a likelihood function falls on the SHASH*_b_*, parameterised byμ,σ,∈, andδ. Model${ℳ}_{3}$is a fitting choice for bothμandσ, as they appear to be related with age. Forμ, we expect that the batch effects do not affect the trajectory of the phenotype, but mainly influence the magnitude. Therefore, the weightswdo not need to be modelled with batch effects, butτdoes. The expected batch effects are not very large, so the non-centered modelling approach (${ℳ}_{2a}$) is a good choice. Model${ℳ}_{3.1.2b}$is decided forμ. We do not expect batch effects inσ, but it does clearly have a relation with age. We can safely choose model$M_{3.1.1}$forσ. The last two parameters,∈andδ, are both assumed to be constant across sites and age. So they are both modelled by${ℳ}_{1}$. To enforce positivity of bothσandδ, we apply a softplus transformation to the outputs of the corresponding models, and to fulfill the constraint discussed inSection 2.1.2, we add a constant toδ.

We now have:

$$\begin{array}{ccccccc} \mu_{n}∼{ℳ}_{3.1.2b} & & n\in \left\{1,\ldots ,N\right\} & & & & \\ \sigma_{n}^{\pm }∼{ℳ}_{3.1.1} & & n\in \left\{1,\ldots ,N\right\} & & & & \\ \sigma_{n}=\text{softplus}\left(\sigma_{n}^{\pm }\right) & & n\in \left\{1,\ldots ,N\right\} & & & & \\ \in ∼{ℳ}_{1} & & & & & & \\ \delta^{\pm }∼{ℳ}_{1} & & & & & & \\ \delta =0.3+\text{softplus}\left(\delta^{\pm }\right) & & & & & & \\ y_{n}∼S_{b}\left(\mu_{n},\sigma_{n},\in ,\delta \right) & & n\in \left\{1,\ldots ,N\right\} & & & & \end{array}$$

## Appendix C Generative Models

In all the generative models in this section,Dis the dimensionality of the basis expansionϕ. In our models, this is taken to be 7. We repeat some earlier equations for quick reference.

$$\begin{array}{l} S_{\in ,\delta }(x)=sinh(\delta sinh^{-1}(x)-\in )\\ S_{\in ,\delta }^{-1}(x)=sinh(sinh^{-1}(x)+\in )/\delta \\ C_{\in ,\delta }(x)=\cosh(\delta \sinh^{-1}(x)-\in )\\ C_{\in ,\delta }=E[X_{\in ,\delta }^{r}]=\frac{1}{2^{r}}\sum_{i=0}^{r}{(}_{i}^{r}){\left(-1\right)}^{i}\exp((r-2i\frac{\in }{\delta }))P((r-2i)/\delta )\\ P(q)=\frac{e^{1/4}}{{\left(8\pi \right)}^{1/2}}(K_{(q+1)/2}(\frac{1}{4})+K_{(q-1)/2}(\frac{1}{4}))\\ {(}^{\eta_{\in ,\delta }})2=m_{\in ,\delta }^{\left(2\right)}-{(}^{m_{\in ,\delta }^{\left(1\right)}})2 \end{array}$$

whereKis a modified Bessel function of the second kind.

### Appendix C.1. Model N

$$\begin{array}{l} {\text{w}}_{\mu }\sim N({\text{w}}_{\mu }|{\text{0}}_{D},{\text{I}}_{D})\\ \mu_{\tau_{\mu }}\sim N(\mu_{\tau_{\mu }}|0,1)\\ \sigma_{\tau_{\mu }}\sim N^{+}(\sigma_{\tau_{\mu }}|1)\\ \nu_{\tau_{\mu_{b}}}\sim N(\nu_{\tau_{\mu_{b}}}-|0,1)\;\;\;\;\;\;\;\;\;\;\;\;\;\;\;\;\;\;\;\;\;\;\;\;\;\;\;\;\;\;\;\;\;\;\;\;\;\;\;\;\;\;\;\;\;\;\;\;\;\;\;\;\;\;\;\;\;\;\;\;\;\;\;\;\;\;\;\;\;\;\;\;\;\;\;\;\;\;\;\;\;\;\;\;\;\;\;\;\;\;\;\;\;\;\;\;\;\;\;\;\;\;\;\;\;\;\;\;\;\;\;b\in \{1,\ldots ,B\}\\ \tau_{\mu_{n}}=\mu_{\tau_{\mu }}+\sigma_{\tau_{\mu }}\nu_{\tau_{\mu_{z_{n}}}}\;\;\;\;\;\;\;\;\;\;\;\;\;\;\;\;\;\;\;\;\;\;\;\;\;\;\;\;\;\;\;\;\;\;\;\;\;\;\;\;\;\;\;\;\;\;\;\;\;\;\;\;\;\;\;\;\;\;\;\;\;\;\;\;\;\;\;\;\;\;\;\;\;\;\;\;\;\;\;\;\;\;\;\;\;\;\;\;\;\;\;\;\;\;\;\;\;\;\;\;\;\;\;\;\;\;\;\;\;\;\;\;n\in \{1,\ldots ,N\}\\ \mu_{n}={\text{w}}_{\mu }^{T}\phi (x_{n})+\tau_{\mu_{n}}\;\;\;\;\;\;\;\;\;\;\;\;\;\;\;\;\;\;\;\;\;\;\;\;\;\;\;\;\;\;\;\;\;\;\;\;\;\;\;\;\;\;\;\;\;\;\;\;\;\;\;\;\;\;\;\;\;\;\;\;\;\;\;\;\;\;\;\;\;\;\;\;\;\;\;\;\;\;\;\;\;\;\;\;\;\;\;\;\;\;\;\;\;\;\;\;\;\;\;\;\;\;\;\;\;\;\;\;\;\;\;\;\;n\in \{1,\ldots ,N\}\\ {\text{w}}_{\sigma^{\pm }}\sim N(\mu_{{\text{w}}_{\sigma^{\pm }}}|{\text{0}}_{D},{\text{I}}_{D})\;\\ \tau_{\sigma^{\pm }}\sim N(\tau_{\sigma^{\pm }}|1,1)\\ \sigma_{n}^{\pm }={\text{w}}_{\sigma }^{T}\phi (x_{n})+\tau_{\sigma^{\pm }}\;\;\;\;\;\;\;\;\;\;\;\;\;\;\;\;\;\;\;\;\;\;\;\;\;\;\;\;\;\;\;\;\;\;\;\;\;\;\;\;\;\;\;\;\;\;\;\;\;\;\;\;\;\;\;\;\;\;\;\;\;\;\;\;\;\;\;\;\;\;\;\;\;\;\;\;\;\;\;\;\;\;\;\;\;\;\;\;\;\;\;\;\;\;\;\;\;\;\;\;\;\;\;\;\;\;\;\;\;\;\;\;n\in \{1,\ldots ,N\}\;\\ \sigma_{n}=\log(1+\exp(\sigma_{n}^{\pm }))=softplus(\sigma_{n}^{\pm })\;\;\;\;\;\;\;\;\;\;\;\;\;\;\;\;\;\;\;\;\;\;\;\;\;\;\;\;\;\;\;\;\;\;\;\;\;\;\;\;\;\;\;\;\;\;\;\;\;\;\;\;\;\;\;\;\;\;\;\;\;\;\;\;\;\;\;\;\;\;\;\;n\in \{1,\ldots ,N\}\\ y_{n}\sim N(y_{n}|\mu_{n},\sigma_{n})\;\;\;\;\;\;\;\;\;\;\;\;\;\;\;\;\;\;\;\;\;\;\;\;\;\;\;\;\;\;\;\;\;\;\;\;\;\;\;\;\;\;\;\;\;\;\;\;\;\;\;\;\;\;\;\;\;\;\;\;\;\;\;\;\;\;\;\;\;\;\;\;\;\;\;\;\;\;\;\;\;\;\;\;\;\;\;\;\;\;\;\;\;\;\;\;\;\;\;\;\;\;\;\;\;\;\;\;\;\;\;\;\;n\in \{1,\ldots ,N\} \end{array}$$

### Appendix C.2. Model $S_{o}$

$$\begin{array}{l} {\text{w}}_{\mu }\sim N({\text{w}}_{\mu }|{\text{0}}_{D}\text{,}{\text{I}}_{D})\\ \mu_{\tau_{\mu }}\sim N(\mu_{\tau_{\mu }}|0,1)\\ \sigma_{\tau_{\mu }}\sim N^{+}(\sigma_{\tau_{\mu }}|1)\\ \nu_{\tau_{\mu_{b}}}\sim N(\nu_{\tau_{\mu_{b}}}-|0,1)\;\;\;\;\;\;\;\;\;\;\;\;\;\;\;\;\;\;\;\;\;\;\;\;\;\;\;\;\;\;\;\;\;\;\;\;\;\;\;\;\;\;\;\;\;\;\;\;\;\;\;\;\;\;\;\;\;\;\;\;\;\;\;\;\;\;\;\;\;\;\;\;\;\;\;\;\;\;\;\;\;\;\;\;\;\;\;\;\;\;\;\;\;\;\;\;\;\;\;\;\;\;\;\;\;\;\;\;\;\;\;b\in \{1,\ldots ,B\}\\ \tau_{\mu_{n}}=\mu_{\tau_{\mu }}+\sigma_{\tau_{\mu }}\nu_{\tau_{\mu_{z_{n}}}}\;\;\;\;\;\;\;\;\;\;\;\;\;\;\;\;\;\;\;\;\;\;\;\;\;\;\;\;\;\;\;\;\;\;\;\;\;\;\;\;\;\;\;\;\;\;\;\;\;\;\;\;\;\;\;\;\;\;\;\;\;\;\;\;\;\;\;\;\;\;\;\;\;\;\;\;\;\;\;\;\;\;\;\;\;\;\;\;\;\;\;\;\;\;\;\;\;\;\;\;\;\;\;\;\;\;\;\;\;\;\;\;n\in \{1,\ldots ,N\}\\ \mu_{n}={\text{w}}_{\mu }^{T}\phi (x_{n})+\tau_{\mu_{n}}\;\;\;\;\;\;\;\;\;\;\;\;\;\;\;\;\;\;\;\;\;\;\;\;\;\;\;\;\;\;\;\;\;\;\;\;\;\;\;\;\;\;\;\;\;\;\;\;\;\;\;\;\;\;\;\;\;\;\;\;\;\;\;\;\;\;\;\;\;\;\;\;\;\;\;\;\;\;\;\;\;\;\;\;\;\;\;\;\;\;\;\;\;\;\;\;\;\;\;\;\;\;\;\;\;\;\;\;\;\;\;\;\;n\in \{1,\ldots ,N\}\\ {\text{w}}_{\sigma^{\pm }}\sim N(\mu_{w_{\sigma^{\pm }}}|{\text{0}}_{D},{\text{I}}_{D})\;\\ \tau_{\sigma^{\pm }}\sim N(\tau_{\sigma^{\pm }}|1,1)\\ \sigma_{n}^{\pm }={\text{w}}_{\sigma }^{T}\phi (x_{n})+\tau_{\sigma^{\pm }}\;\;\;\;\;\;\;\;\;\;\;\;\;\;\;\;\;\;\;\;\;\;\;\;\;\;\;\;\;\;\;\;\;\;\;\;\;\;\;\;\;\;\;\;\;\;\;\;\;\;\;\;\;\;\;\;\;\;\;\;\;\;\;\;\;\;\;\;\;\;\;\;\;\;\;\;\;\;\;\;\;\;\;\;\;\;\;\;\;\;\;\;\;\;\;\;\;\;\;\;\;\;\;\;\;\;\;\;\;\;\;\;n\in \{1,\ldots ,N\}\;\\ \sigma_{n}=\log(1+\exp(\sigma_{n}^{\pm }))=softplus(\sigma_{n}^{\pm })\;\;\;\;\;\;\;\;\;\;\;\;\;\;\;\;\;\;\;\;\;\;\;\;\;\;\;\;\;\;\;\;\;\;\;\;\;\;\;\;\;\;\;\;\;\;\;\;\;\;\;\;\;\;\;\;\;\;\;\;\;\;\;\;\;\;\;\;\;\;\;\;n\in \{1,\ldots ,N\}\\ \in \;\sim N(\in |0,1)\\ \delta^{\pm }\sim N(\delta^{\pm }|1,1)\\ \delta =log(1+exp(10\cdot \delta^{\pm }))/10=softplus(10\cdot \delta^{\pm })/10\\ y_{n}\sim S_{o}(y_{n}|\mu_{n},\sigma_{n},\in ,\delta )=\frac{\delta C_{\in ,\delta }(\Gamma )}{\sqrt{1+x^{2}}}\frac{1}{\sqrt{2\pi }}\exp\left(\frac{-S_{\in ,\delta }{\left(\Gamma \right)}^{2}}{2}\right)\sigma_{n}^{-1}\;\;\;\;\;\;\;\;\;\;\;\;n\in \{1,\ldots ,N\}\;\; \end{array}$$

where

$$\Gamma =\left(y_{n}-\mu_{n}\right)\sigma_{n}^{-1}$$

### Appendix C.3. Model $S_{b1}$

$$\begin{array}{l} {\text{w}}_{\mu }\sim N({\text{w}}_{\mu }|{\text{0}}_{D},{\text{I}}_{D})\\ \mu_{\tau_{\mu }}\sim N(\mu_{\tau_{\mu }}|\text{0},1)\\ \sigma_{\tau_{\mu }}\sim N^{+}(\sigma_{\tau_{\mu }}|1)\\ \nu_{\tau_{\mu_{b}}}\sim N(\nu_{\tau_{\mu_{b}}}-|\text{0},1)\;\;\;\;\;\;\;\;\;\;\;\;\;\;\;\;\;\;\;\;\;\;\;\;\;\;\;\;\;\;\;\;\;\;\;\;\;\;\;\;\;\;\;\;\;\;\;\;\;\;\;\;\;\;\;\;\;\;\;\;\;\;\;\;\;\;\;\;\;\;\;\;\;\;\;\;\;\;\;\;\;\;\;\;\;\;\;\;\;\;\;\;\;\;\;\;\;\;\;\;\;\;\;\;\;\;\;\;\;\;\;\;\;\;\;\;\;\;\;\;\;\;\;\;b\in \{1,\ldots ,B\}\\ \tau_{\mu_{n}}=\mu_{\tau_{\mu }}+\sigma_{\tau_{\mu }}\nu_{\tau_{\mu_{z_{n}}}}\;\;\;\;\;\;\;\;\;\;\;\;\;\;\;\;\;\;\;\;\;\;\;\;\;\;\;\;\;\;\;\;\;\;\;\;\;\;\;\;\;\;\;\;\;\;\;\;\;\;\;\;\;\;\;\;\;\;\;\;\;\;\;\;\;\;\;\;\;\;\;\;\;\;\;\;\;\;\;\;\;\;\;\;\;\;\;\;\;\;\;\;\;\;\;\;\;\;\;\;\;\;\;\;\;\;\;\;\;\;\;\;\;\;\;\;\;\;\;\;\;\;\;n\in \{1,\ldots ,N\}\\ \mu_{n}={\text{w}}_{\mu }^{T}\phi (x_{n})+\tau_{\mu_{n}}\;\;\;\;\;\;\;\;\;\;\;\;\;\;\;\;\;\;\;\;\;\;\;\;\;\;\;\;\;\;\;\;\;\;\;\;\;\;\;\;\;\;\;\;\;\;\;\;\;\;\;\;\;\;\;\;\;\;\;\;\;\;\;\;\;\;\;\;\;\;\;\;\;\;\;\;\;\;\;\;\;\;\;\;\;\;\;\;\;\;\;\;\;\;\;\;\;\;\;\;\;\;\;\;\;\;\;\;\;\;\;\;\;\;\;\;\;\;\;\;\;\;\;\;\;n\in \{1,\ldots ,N\}\\ {\text{w}}_{\sigma^{\pm }}\sim N(\mu_{{\text{w}}_{\sigma^{\pm }}}|{\text{0}}_{D},{\text{I}}_{D})\;\\ \tau_{\sigma^{\pm }}\sim N(\tau_{\sigma^{\pm }}|1,1)\\ \sigma_{n}^{\pm }={\text{w}}_{\sigma }^{T}\phi (x_{n})+\tau_{\sigma^{\pm }}\;\;\;\;\;\;\;\;\;\;\;\;\;\;\;\;\;\;\;\;\;\;\;\;\;\;\;\;\;\;\;\;\;\;\;\;\;\;\;\;\;\;\;\;\;\;\;\;\;\;\;\;\;\;\;\;\;\;\;\;\;\;\;\;\;\;\;\;\;\;\;\;\;\;\;\;\;\;\;\;\;\;\;\;\;\;\;\;\;\;\;\;\;\;\;\;\;\;\;\;\;\;\;\;\;\;\;\;\;\;\;\;\;\;\;\;\;\;\;\;\;\;\;\;n\in \{1,\ldots ,N\}\;\\ \sigma_{n}=\log(1+\exp(\sigma_{n}^{\pm }))=softplus(\sigma_{n}^{\pm })\;\;\;\;\;\;\;\;\;\;\;\;\;\;\;\;\;\;\;\;\;\;\;\;\;\;\;\;\;\;\;\;\;\;\;\;\;\;\;\;\;\;\;\;\;\;\;\;\;\;\;\;\;\;\;\;\;\;\;\;\;\;\;\;\;\;\;\;\;\;\;\;\;\;\;\;\;\;\;\;\;\;\;\;n\in \{1,\ldots ,N\}\\ \in \;\sim N(\in |\text{0},1)\\ \delta^{\pm }\sim N(\delta^{\pm }|1,1)\\ \delta =0.3+log(1+exp(10\cdot \delta^{\pm }))/10=0.3+softplus(10\cdot \delta^{\pm })/10\\ y_{n}\sim S_{b}(y_{n}|\mu_{n},\sigma_{n},\in ,\delta )=\frac{\delta C_{\in ,\delta }(\Gamma )}{\sqrt{1+x^{2}}}\frac{1}{\sqrt{2\pi }}\exp\left(\frac{-S_{\in ,\delta }{\left(\Gamma \right)}^{2}}{2}\right)\eta_{\in ,\delta }\sigma_{n}^{-1}\;\;\;\;\;\;\;\;\;\;\;\;\;n\in \{1,\ldots ,N\}\;\; \end{array}$$

where

$$\Gamma =\left(\left(y_{n}\eta_{\in ,\delta }+m_{\in ,\delta }^{\left(1\right)}\right)-\mu_{n}\right)\sigma_{n}^{-1}$$

### Appendix C.4 Model $S_{b2}$

$$\begin{array}{l} {\text{w}}_{\mu }\sim N({\text{w}}_{\mu }|{\text{0}}_{D},{\text{I}}_{D})\\ \mu_{\tau_{\mu }}\sim N(\mu_{\tau_{\mu }}|\text{0},1)\\ \sigma_{\tau_{\mu }}\sim N^{+}(\sigma_{\tau_{\mu }}|1)\\ \nu_{\tau_{\mu_{b}}}\sim N(\nu_{\tau_{\mu_{b}}}|\text{0},1)\;\;\;\;\;\;\;\;\;\;\;\;\;\;\;\;\;\;\;\;\;\;\;\;\;\;\;\;\;\;\;\;\;\;\;\;\;\;\;\;\;\;\;\;\;\;\;\;\;\;\;\;\;\;\;\;\;\;\;\;\;\;\;\;\;\;\;\;\;\;\;\;\;\;\;\;\;\;\;\;\;\;\;\;\;\;\;\;\;\;\;\;\;\;\;\;\;\;\;\;\;\;\;\;\;\;\;\;\;\;\;\;\;\;\;\;\;\;\;\;\;\;\;\;\;\;\;\;\;\;\;\;\;\;\;\;\;\;\;\;\;\;\;\;b\in \{1,\ldots ,B\}\\ \tau_{\mu_{n}}=\mu_{\tau_{n}}+\sigma_{\tau_{\mu }}\nu_{\tau_{\mu_{z_{n}}}}\;\;\;\;\;\;\;\;\;\;\;\;\;\;\;\;\;\;\;\;\;\;\;\;\;\;\;\;\;\;\;\;\;\;\;\;\;\;\;\;\;\;\;\;\;\;\;\;\;\;\;\;\;\;\;\;\;\;\;\;\;\;\;\;\;\;\;\;\;\;\;\;\;\;\;\;\;\;\;\;\;\;\;\;\;\;\;\;\;\;\;\;\;\;\;\;\;\;\;\;\;\;\;\;\;\;\;\;\;\;\;\;\;\;\;\;\;\;\;\;\;\;\;\;\;\;\;\;\;\;\;\;\;\;\;\;\;\;\;\;n\in \{1\ldots ,N\}\\ \mu_{n}={\text{w}}_{\mu }^{T}\phi (x_{n})+\tau_{\mu_{n}}\;\;\;\;\;\;\;\;\;\;\;\;\;\;\;\;\;\;\;\;\;\;\;\;\;\;\;\;\;\;\;\;\;\;\;\;\;\;\;\;\;\;\;\;\;\;\;\;\;\;\;\;\;\;\;\;\;\;\;\;\;\;\;\;\;\;\;\;\;\;\;\;\;\;\;\;\;\;\;\;\;\;\;\;\;\;\;\;\;\;\;\;\;\;\;\;\;\;\;\;\;\;\;\;\;\;\;\;\;\;\;\;\;\;\;\;\;\;\;\;\;\;\;\;\;\;\;\;\;\;\;\;\;\;\;\;\;\;\;\;\;n\in \{1\ldots ,N\}\\ {\text{w}}_{\sigma^{\pm }}\sim N(\mu_{{\text{w}}_{\sigma^{\pm }}}|{\text{0}}_{D},{\text{I}}_{D})\\ \tau_{\sigma^{\pm }}\sim N(\tau_{\sigma^{\pm }}|1,1)\\ \sigma_{n}^{\pm }={\text{w}}_{\sigma }^{T}\phi (x_{n})+\tau_{\sigma^{\pm }}\;\;\;\;\;\;\;\;\;\;\;\;\;\;\;\;\;\;\;\;\;\;\;\;\;\;\;\;\;\;\;\;\;\;\;\;\;\;\;\;\;\;\;\;\;\;\;\;\;\;\;\;\;\;\;\;\;\;\;\;\;\;\;\;\;\;\;\;\;\;\;\;\;\;\;\;\;\;\;\;\;\;\;\;\;\;\;\;\;\;\;\;\;\;\;\;\;\;\;\;\;\;\;\;\;\;\;\;\;\;\;\;\;\;\;\;\;\;\;\;\;\;\;\;\;\;\;\;\;\;\;\;\;\;\;\;\;\;\;\;\;n\in \{1\ldots ,N\}\\ \sigma_{n}=\log(1+\exp(\sigma_{n}^{\pm }))=softplus(\sigma_{n}^{\pm })\;\;\;\;\;\;\;\;\;\;\;\;\;\;\;\;\;\;\;\;\;\;\;\;\;\;\;\;\;\;\;\;\;\;\;\;\;\;\;\;\;\;\;\;\;\;\;\;\;\;\;\;\;\;\;\;\;\;\;\;\;\;\;\;\;\;\;\;\;\;\;\;\;\;\;\;\;\;\;\;\;\;\;\;\;\;\;\;\;\;\;\;\;\;\;\;\;\;\;\;\;\;n\in \{1\ldots ,N\}\\ {\text{w}}_{\in }\sim N({\text{w}}_{\in }|{\text{0}}_{D},0.2\cdot {\text{I}}_{D})\\ \tau_{\in }\sim N(\tau_{\in }|\text{0},0.2)\\ \in_{n}={\text{w}}_{\sigma }^{T}\phi (x_{n})+\tau_{\in }\;\;\;\;\;\;\;\;\;\;\;\;\;\;\;\;\;\;\;\;\;\;\;\;\;\;\;\;\;\;\;\;\;\;\;\;\;\;\;\;\;\;\;\;\;\;\;\;\;\;\;\;\;\;\;\;\;\;\;\;\;\;\;\;\;\;\;\;\;\;\;\;\;\;\;\;\;\;\;\;\;\;\;\;\;\;\;\;\;\;\;\;\;\;\;\;\;\;\;\;\;\;\;\;\;\;\;\;\;\;\;\;\;\;\;\;\;\;\;\;\;\;\;\;\;\;\;\;\;\;\;\;\;\;\;\;\;\;\;\;\;\;\;\;\;\;n\in \{1\ldots ,N\}\;\\ {\text{w}}_{\delta^{\pm }}\sim N({\text{w}}_{\delta^{\pm }}|{\text{0}}_{D},0.2\cdot {\text{I}}_{D})\\ \tau_{\delta^{\pm }}\sim N(\tau_{\pm }|\text{0},0.3)\\ \delta_{n}^{\pm }={\text{w}}_{\delta^{\pm }}T\phi (x_{n})+\tau_{\delta^{\pm }}\;\;\;\;\;\;\;\;\;\;\;\;\;\;\;\;\;\;\;\;\;\;\;\;\;\;\;\;\;\;\;\;\;\;\;\;\;\;\;\;\;\;\;\;\;\;\;\;\;\;\;\;\;\;\;\;\;\;\;\;\;\;\;\;\;\;\;\;\;\;\;\;\;\;\;\;\;\;\;\;\;\;\;\;\;\;\;\;\;\;\;\;\;\;\;\;\;\;\;\;\;\;\;\;\;\;\;\;\;\;\;\;\;\;\;\;\;\;\;\;\;\;\;\;\;\;\;\;\;\;\;\;\;\;\;\;\;n\in \{1\ldots ,N\}\;\\ \delta_{n}=0.3+\log(1+\exp(10\cdot \delta_{n}^{\pm }))/10=0.3+softplus(10\cdot \delta_{n}^{\pm })/10\\ y_{n}\sim S_{b}(y_{n}|\mu_{n},\sigma_{n},\in_{n},\delta_{n})=\frac{\delta C_{\in_{n},\delta_{n}}(\Gamma )}{\sqrt{1+x^{2}}}\frac{1}{\sqrt{2\pi }}\exp\left(\frac{-S_{\in_{n},\delta_{n}}{\left(\Gamma \right)}^{2}}{2}\right)\eta_{\in_{n},\delta_{n}}\sigma_{n}^{-1}\;\;\;\;\;\;\;\;\;\;\;n\in \{1,\ldots ,N\} \end{array}$$

where

$$\Gamma =\left(\left(y_{n}\eta_{\in_{n}\delta_{n}}+m_{\in_{n},\delta_{n}}^{\left(1\right)}\right)-\mu_{n}\right)\sigma_{n}^{-1}$$

## Appendix D MCMC Sampling In A Nutshell

Uncertainty quantification ideally accounts for all uncertainty in the model parameters, but is often hard to quantify exactly. MCMC approximates this uncertainty by sampling from the desired distribution. Given a generative model like above, Bayes’ rule gives us an expression for the posterior over our model parameters:

$$p(\theta |D)=\frac{p(D|\theta )p(\theta )}{p(D)},$$
(D. 1)

which we then use to compute the expectation of a functionf:

$$E_{\theta ∼p(\theta |D)}\left[f\left(\theta \right)\right]=\int_{\theta }f\left(\theta \right)p(\theta |D)d\theta$$
(D. 2)

For instance, in normative modelling,fcould return the deviation score of some data point. The expectation above gives us an estimate in which all the uncertainty in the model parameters is captured. Unfortunately, the integral inEquation D.2is intractable, and the denominator inEquation D.1,p(D), contains an intractable integral as well:

$$p(D)=\int_{\theta^{\prime }}p(D|\theta^{\prime })p(\theta^{\prime })d\theta^{\prime }$$
(D. 3)

In practice, it is impossible to determinep(θ|D)exactly, unless the prior is conjugate to the likelihood. MCMC samplers use a number of different methods to retrieve pseudo-random samples fromp(θ|D), without ever needing to analyse the integral inEquation D.3. With enough of these samples in hand, we use the following result:

$$\begin{array}{c} \theta ∼p\left(\theta \text{|}D\right),\\ \underset{N\to \infty }{\text{lim}}\frac{1}{N}\overset{N}{\underset{i=1}{\sum }}f\left(\theta_{i}\right)=\int_{\theta }f\left(\theta \right)p(\theta |D)d\theta =E_{\theta ∼p(\theta |D)}\left[f\left(\theta \right)\right] \end{array}$$

to approximate our desired expectation.

## Appendix E Maximum-a-Posteriori (MAP) Approximation

Instead of doing an MCMC estimation, which is quite computationally expensive, another method is to find the MAP, and to compute the desired function based on that point.

$$\theta^{\text{MAP}}=\text{arg}\underset{\theta }{\text{max}}p(\theta |D)$$

So in other words, the MAP estimate is just that point in parameter space that maximises the posterior. It can be found using gradient-based methods. Using the MAP-estimate is not the most principled method, because it does not account for the uncertainty in the parameters. For instance, the MAP estimate will always return a singleθ, although the posterior could by multimodal, or contain large flat optima, which would justify multiple validθ’s. However, the computational gains may be worth the loss of a representation of uncertainty.
